# Modelling and mutational analysis of *Aspergillus nidulans* UreA, a member of the subfamily of urea/H^+^ transporters in fungi and plants

**DOI:** 10.1098/rsob.140070

**Published:** 2014-06-25

**Authors:** Manuel Sanguinetti, Sotiris Amillis, Sergio Pantano, Claudio Scazzocchio, Ana Ramón

**Affiliations:** 1 Sección Bioquímica, Departamento de Biología Celular y Molecular, Facultad de Ciencias, Universidad de la República, Montevideo, Uruguay; 2 Faculty of Biology, Department of Botany, University of Athens, Athens, Greece; 3 Biomolecular Simulations Group, Institut Pasteur de Montevideo, Montevideo, Uruguay; 4 Institut de Génétique et Microbiologie, Université Paris-Sud, Orsay, France; 5 Department of Microbiology, Imperial College London, London, UK

**Keywords:** sodium : solute symporter-family, structure–function relationships, permease

## Abstract

We present the first account of the structure–function relationships of a protein of the subfamily of urea/H^+^ membrane transporters of fungi and plants, using *Aspergillus nidulans* UreA as a study model. Based on the crystal structures of the *Vibrio parahaemolyticus* sodium/galactose symporter (vSGLT) and of the Nucleobase-Cation-Symport-1 benzylhydantoin transporter from *Microbacterium liquefaciens* (Mhp1), we constructed a three-dimensional model of UreA which, combined with site-directed and classical random mutagenesis, led to the identification of amino acids important for UreA function. Our approach allowed us to suggest roles for these residues in the binding, recognition and translocation of urea, and in the sorting of UreA to the membrane. Residues W82, Y106, A110, T133, N275, D286, Y388, Y437 and S446, located in transmembrane helixes 2, 3, 7 and 11, were found to be involved in the binding, recognition and/or translocation of urea and the sorting of UreA to the membrane. Y106, A110, T133 and Y437 seem to play a role in substrate selectivity, while S446 is necessary for proper sorting of UreA to the membrane. Other amino acids identified by random classical mutagenesis (G99, R141, A163, G168 and P639) may be important for the basic transporter's structure, its proper folding or its correct traffic to the membrane.

## 2. Introduction

Urea is a readily available nitrogen source for bacteria, fungi and plants, occurring in nature as a product of animal, plant and microbial metabolism of nitrogenous compounds [1–6]. Additionally, together with ammonium nitrate it is one of the most widely used nitrogen-based fertilizers (http://espere.mpch-mainz.mpg.de/documents/ACCENT/Edition08/texts%20material/english/fertilizer_usage_2001.pdf [7]). The internalization of this highly polar molecule necessitates specialized cellular transport systems. Fungi and plants possess a specific urea permease which is structurally and mechanistically different from animal or bacterial transporters, and which constitutes a subfamily of H^+^-symporters within the sodium : solute symporter (SSS) superfamily. The latter is composed by hundreds of members, present in both eukaryotic and prokaryotic organisms, which usually function as Na^+^-cotransporters of a variety of substrates, such as sugars, vitamins, amino acids, inorganic ions, etc. (http://www.tcdb.org [8]).

Members of this urea/H^+^ symporters group have been functionally characterized in the fungi *Saccharomyces cerevisiae* (ScDUR3 [9]), *Paxillus involutus* (PiDUR3 [10]), *Aspergillus nidulans* (UreA [11]) and *Candida albicans* (CaDUR3 [12]), and in the plants *Arabidopsis thaliana* (AtDUR3 [13,14]) and *Oryza sativa* (OsDUR3 [15]). They share 35–74% of pairwise sequence identity and most of them include 14–15 predicted α-helical transmembrane segments (TMS). In *A. nidulans*, the urea transporter UreA has been identified as the only high-affinity urea transport system in this organism [11]. A second transport system may mediate facilitated diffusion at high urea concentrations (more than or equal to 3 mM) [11,16]. UreA is predicted to have 15 TMS [11]. Kinetic studies have established that it transports urea with a *K_m_* of 26 μM, transport being inhibited by 2-thiourea (a urea toxic analogue) and acetamide. The transcription of the cognate gene, *ureA*, depends on the AreA GATA factor; it is repressible by ammonium and is not inducible by urea or its precursors. As for other permease genes, *ureA* expression is upregulated during the isotropic growth phase of the conidiospores, but, at variance with most permeases involved in the uptake of nitrogen sources, the gene is still under strict nitrogen catabolite repression in this phase of growth. A post-translational regulatory mechanism operates on UreA, which is endocytosed in response to ammonium. Putative orthologues of UreA are present in all sequenced Aspergilli. Moreover, three other paralogues of UreA have been identified in *A. nidulans* and are also present in other Aspergilli, though not all species genomes include all paralogues. These proteins may be low-affinity urea transporters, as *ureA* deleted strains (*ureA*Δ) only show residual growth when urea is used as sole nitrogen source. This fact has been attributed to a secondary energy-independent transport system, as mentioned above, or otherwise may be due to paralogues with different and unknown specificity, but which may residually transport urea [11].

The complex and hydrophobic nature of transporters makes their purification and crystallization difficult. However, three-dimensional modelling on solved crystal structures combined with random and directed mutagenesis can shed light on different aspects of a transporter's conformation and function, as illustrated clearly for the first time with homologues of the lactose permease LacY [17–22] and of the neurotransmitter-transporter prototype LeuT [23–26]. In the *A. nidulans* system, the power of this kind of approach has been illustrated by the thorough dissection of the high-affinity/high-capacity xanthine/uric acid transporter UapA [27,28].

The crystal structure of a member of the SSS superfamily, the sodium/galactose symporter of *Vibrio parahaemolyticus*, vSGLT, has been solved at approximately 3 Å resolution [30,48]. It shows a five-helix inverted repeat motif (the 5-HIR-fold), also found in other bacterial transporters that do not share any sequence similarity and that transport a wide variety of substrates, like LeuT [31–33], Mhp1[29,34] and BetP [35,36] among others ([37,38] and references therein). A ‘rocking-bundle’ mechanism has been proposed for transporters bearing this structure, which implies a substrate-induced alternation between inward- and outward-facing hydrophilic cavities, and the opening and closing of specific gating amino acids [26,39,40]. We present below a structure–function analysis of UreA, based on comparative modelling, and random and site-directed mutagenesis. We identify mutations affecting substrate binding and/or translocation and membrane sorting of the transporter. This constitutes the first such analysis of a transporter of this family.

## 3. Results

### 3.1. Rational design of site-directed mutagenesis and construction of mutant strains

**Figure 1.**
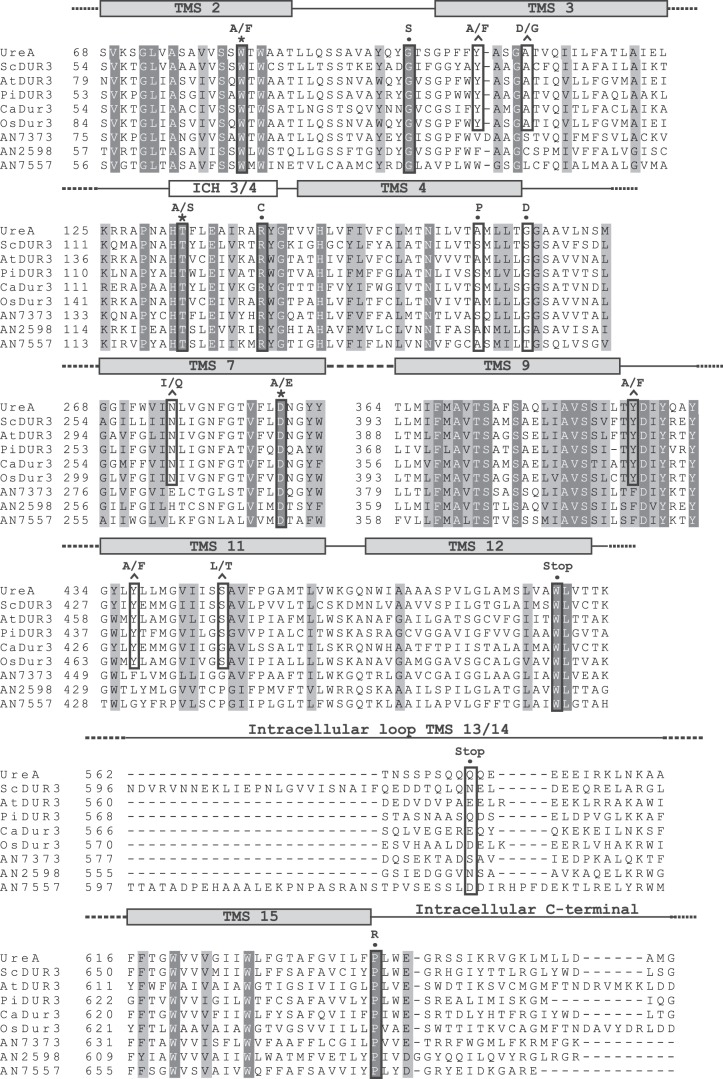
Multiple sequence alignment of UreA and homologues. Aligned sequences include *A. nidulans* UreA (GI: 67516273), characterized orthologues in fungi and plants—ScDur3 of *S. cerevisiae* (GI: 51013791), AtDur3 of *A. thaliana* (GI: 9758728), PiDur3 of *P. involutus* (sequence kindly provided by Morel *et al.* [10]), CaDur3 of *C. albicans* (GI: 68484979) and OsDur3 of *O. sativa* (GI: 115483686)—and UreA paralogues AN7373 (GI: 259483267), AN2598 (GI: 259488035) and AN7557 (GI: 67901140). Putative TMSs and the intracellular helix between TMS3 and TMS4 (ICH3/4) are represented by grey and white rectangles above the sequence, respectively. For space reasons, only those TMSs, ICHs and loops including mutated residues are shown; omitted segments are represented by dashed lines. Fully conserved amino acids are shaded in black, and structurally conserved amino acids are shaded in grey. The mutations obtained in this work, whether by classical or directed mutagenesis, are boxed in black; residues affected by directed mutagenesis and conserved in UreA orthologues but not in its paralogues are denoted by a circumflex above the sequence, while those affecting amino acids conserved in all UreA homologues are denoted by an asterisk. The aligned residues affected by classical mutagenesis are denoted by a filled circle.

Following our previous characterization of UreA [11], we aimed to identify key residues involved in urea transport. We assumed that important residues for transport activity would be conserved in functionally characterized UreA orthologues (ScDUR3, CaDUR3, AtDUR3, PiDUR3 and OsDUR3) but not in its paralogue proteins (ANID_07373.1, ANID_02598.1 and ANID_07557.1), which do not significantly transport urea. Therefore, we aligned these eight homologous proteins against the primary sequence of UreA (figure 1). Residues fulfilling the above-mentioned criteria are Y106, A110, N275, A374, Y388, Y437 and S446. Following the predicted secondary structure of UreA [11], these residues are estimated to be located, respectively, in transmembrane helixes 3 (Y106, A110), 7 (N275), 9 (A374) and 11 (Y437 and S446), while Y388 is located immediately after TMS9 (figure 1).

**Figure 2.**
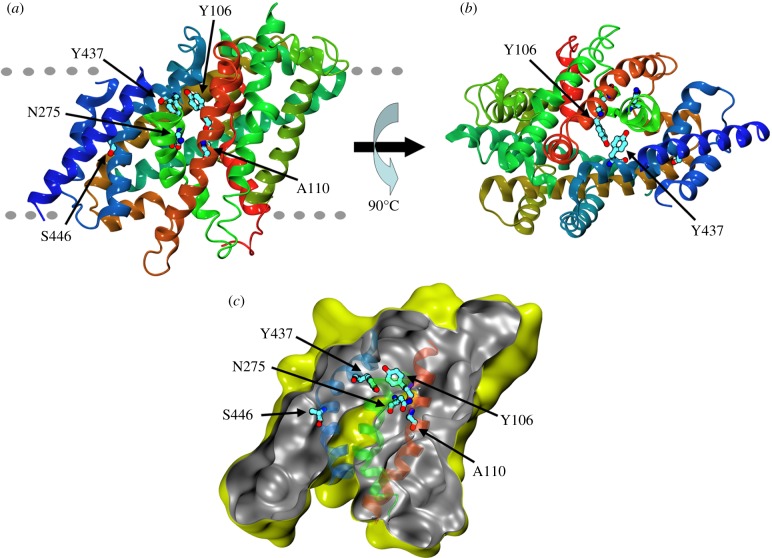
Molecular model of UreA in the inward-facing, closed conformation. (*a*) Secondary structure elements are coloured by sequence number going continuously from red (amino terminal) to blue (carboxy terminal). The rough position of the membrane is indicated by the grey dotted lines with the extracellular surface on the upper part of the figure. (*b*) Same as (*a*), viewed from outside the membrane and rotated 90° along the membrane plane. (*c*) Solvent accessible surface calculated with a probe radius of 0.2 nm. The slice on the surface is taken perpendicularly to the membrane to show the inward-facing cavity. External and internal sides of the surface are yellow and grey, respectively. For clarity, only the semitransparent cartoon representation of helixes 3, 7 and 11 is shown, coloured as in (*a*).

We sought to construct a model of UreA comprising amino acids 64–525, spanning TMSs 2–13 (electronic supplementary material, figure S1), based on the structure of vSGLT of *V. parahaemolyticus* [30]. This structure is representative of the transporter in a closed state with an inward-facing cavity. It must be noted that, owing to the low sequence identity between the crystallized protein and UreA (see Experimental procedures), the reliability of the model is limited to the positioning of secondary structure elements and rough location of amino acids. The predicted TMSs of UreA modelled on vSGLT and Mhp1 (see below) are shown in the electronic supplementary material, figure S1. TMSs 3, 7 and 11 are cognate to the segments where, in vSGLT, key residues of the substrate-binding site and translocation pathway are located. Thus, five out of seven conserved residues in characterized urea transporters are located in these putatively important regions. In particular, Y106 and Y437 are predicted to face each other at the bottom of an inward-facing cavity (figure 2*a*,*b*), while A110 lines the cavity, located one helix-turn above Y106 (figure 2*a*,*c*). N275 and S446 are located in helixes 7 and 11, which line the cavity, though neither of them faces it. The localization of residues Y106, A110, N275, S446 and Y437 supports their putative functional role, and thus they were selected for site-directed mutagenesis. Residues A374 and Y388, which are not predicted to localize to any of these helixes, were not included among the residues we chose to mutate.

**Figure 3.**
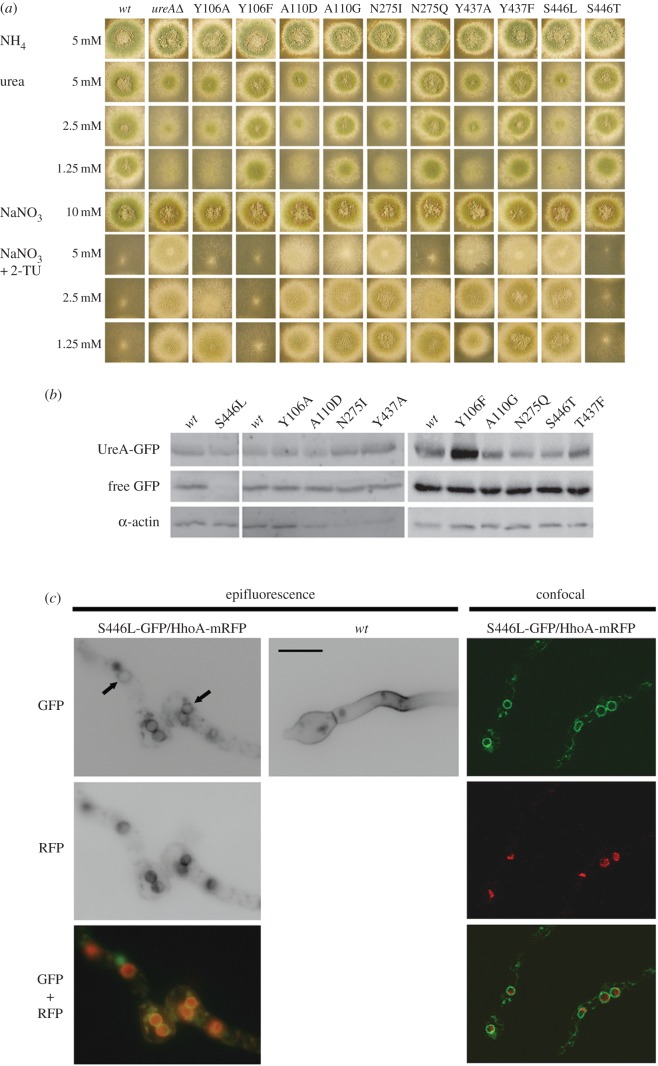
Characterization of strains bearing mutations in residues conserved in functionally characterized UreA orthologues. (*a*) Growth phenotypes of mutant UreA strains at 37°C on urea as nitrogen source or on 2-thiourea with NaNO_3_ 10 mM as nitrogen source to test resistance to the analogue. Growth on ammonium 5 mM and nitrate 10 mM are used as controls. A *wt* and a *ureA*Δ strain are shown as positive and negative controls, respectively. Similar results were obtained at 25°C (not shown). (*b*) Western blot analysis of total protein extracts of UreA–GFP mutants probed with anti-GFP antibody. Cultures were grown for 14–16 h at 25°C in derepressed conditions (proline as sole nitrogen source). The low mobility band corresponds to intact UreA–GFP and the high mobility band to free GFP (see text). Antibody against actin was used as an internal control of loading. (*c*) Epifluorescence and confocal microscopy of mutant bearing the S446L mutation grown in derepressing conditions, showing the retention of the UreA–GFP fusion in the ER, seen as perinuclear rings (indicated by arrows). Upper panels show the localization of the GFP signal. Wild-type UreA–GFP localization is shown as control. Middle panels show the localization to the nucleus of a histone H1 (HhoA)–mRFP fusion. Lower panels display the colocalization of GFP and RFP signals. Scale bar, 10 µm.

The residues discussed above were mutated as indicated in figure 1. In all cases, genomic DNA from a strain carrying a *wild-type* (*wt*) UreA–GFP fusion was used as the template, so that the subcellular localization of the resulting mutant–GFP fusions could be immediately assessed. Single copy transformants integrated to the *ureA locus* were obtained by transforming a *ureA*Δ strain, as described in Experimental procedures. All mutants were tested for utilization of urea as nitrogen source, resistance to 2-thiourea and kinetics of urea uptake as described in Experimental procedures. Non-conservative changes yielded mutations Y106A, A110D, N275I, Y437A and S446L. When grown on urea as sole nitrogen source at concentrations of 0.625–5 mM, all substitutions described above result in phenotypes identical to those of the *ureA*Δ strain [11], showing residual growth similar to that seen in the absence of any nitrogen source, both at 37°C (figure 3*a*) and at 25°C (not shown). The resistance to 2-thiourea seen at concentrations of 0.625–5 mM is also comparable to that observed for the *ureA*Δ strain except for Y106A (see below). Western blots show that this phenotype is not due to the absence of the protein (figure 3*b*).

In accordance with growth tests, ^14^C-urea initial apparent uptake rates for strains carrying mutations A110D, N275I, S446L and Y437A were less than or equal to 2% of the *wt* (table 1). Mutation Y106A results in partial impairment of ^14^C-urea uptake rates (approx. 14% of that of the *wt*). This is in agreement with growth tests on 2-thiourea, where sensitivity can be observed at concentrations more than or equal to 2.5 mM (figure 3*a*). Substrate binding was assessed by calculating *K_m_* values for urea and *K_i_* values for 2-thiourea, acetamide and guanidine. The latter is a structural analogue of urea that only binds UreA at concentrations more than or equal to 3 mM. Y106A increases the *K_m_* for urea and the *K_i_* for 2-thiourea and acetamide by a factor of approximately 2 (table 1), while the *K_i_* for guanidine is diminished. It must be taken into account that phenotypic differences in growth plates can only be detected when *K_m_*_/*i*_ values are higher than the substrate concentration used in growth tests, as is the case for Y106A.

**Table 1.** Summary of UreA mutations obtained by site-directed mutagenesis and their effect on subcellular localization and functionality. TMS, transmembrane segment; ICH3/4, intracellular helix between TMS3 and TMS4; M, localization to the membrane; ER, retention in the endoplasmic reticulum; n.a., not applicable. +++, growth phenotype on urea as that of the *wt*; −−−, growth phenotype on urea as that of the *ureA*Δ; ++−, +−−, intermediate growth phenotypes between that of the *wt* and that of the *ureA*Δ strain. *V* (%): initial uptake rates, expressed as % of the *wt* in pmoles min^−1^/10^7^ conidiospores.

allele	location in protein^a^	subcellular localization	growth on urea	growth on 2-thiourea	*V* (%)	urea *K_m_* (µM)	2-thiourea *K_i_* (µM)	acetamide *K_i_* (µM)	guanidine *K_i_* (µM)
*wt*		M	+++	−−−	100	26 ± 2	528 ± 42	237 ± 14	>3000
*ureA*Δ		n.a.	−−−	+++	<2				
Y106A	TMS3	M	−−−	+−−	14 ± 3	52 ± 12	978 ± 73	434 ± 24	1650 ± 130
Y106F		M	+++	−−−	29 ± 6	43 ± 4	591 ± 55	312 ± 17	1900 ± 175
A110D	TMS3	M	−−−	++−	<2				
A110G		M	++−	++−	42 ± 5	92 ± 6	950 ± 84	495 ± 29	>3000
N275I	TMS7	M	−−−	+++	<2				
N275Q		M	+++	+−−	91 ± 8	33 ± 4	1035 ± 92	352 ± 26	>3000
Y437A	TMS11	M	−−−	++−	<2				
Y437F		M	+++	++−	46 ± 7	88 ± 9	1370 ± 107	102 ± 15	>3000
S446L	TMS11	ER	−−−	+++	<2				
S446T		M	+++	−−−	102 ± 12	24 ± 3	562 ± 37	291 ± 29	>3000
W82A	TMS2	M	−−−	++−	<2				
W82F		M	+−−	−−−	50 ± 8	23 ± 5	447 ± 24	198 ± 12	>2500
T133A	ICH3/4	M	−−−	++−	<2				
T133S		M	+++	+−−	72 ± 9	48 ± 3	693 ± 38	316 ± 20	>3000
D286A	TMS7	M	−−−	+++	<2				
D286E		M	+++	−−−	102 ± 10	29 ± 5	555 ± 31	289 ± 24	>3000
Y388A	TMS9	M	−−−	++−	<2				
Y388F		M	+++	−−−	100 ± 5	25 ± 4	602 ± 44	226 ± 23	>3000

^a^According to the constructed three-dimensional models.

Conservative changes at the same selected positions were carried out to yield mutations Y106F, A110G, N275Q, Y437F and S446T (figure 1). When Y106 is changed to Phe, the mutant strain shows growth phenotypes on urea and 2-thiourea that are practically indistinguishable from the *wt*. ^14^C-urea transport assays provide a *K_m_* for urea that is 1.6 times higher than those of the *wt*. The *K_i_* value for acetamide is slightly higher than that of the *wt* (312 ± 17 µM, against 237 ± 14 µM in the *wt*; table 1), while, as observed for Y106A, the *K_i_* for guanidine is diminished. The initial urea uptake rate of mutant Y106F reaches only approximately 30% of that of the *wt*, which suggests that even if substrate affinity is only marginally affected, its transport across the membrane is further impaired. These differences are not detectable in growth tests.

Mutations A110G, Y437F and N275Q affect similarly growth on urea and sensitivity to 2-thiourea. On urea, N275Q and Y437F are almost indistinguishable from the *wt* (figure 3*a*), whereas A110G shows a very subtle growth defect. The initial uptake rates of mutants Y437F and N275Q are approximately 50% and approximately 90%, respectively, as compared with the *wt*, while in the case of mutant A110G, the initial uptake rate attains approximately 40% of the *wt*. Urea *K_m_* found in strains carrying these mutations are 92 ± 6 µM for A110G; 33 ± 4 µM for N275Q and 88 ± 9 µM for Y437F (table 1), compared with a value of 26 ± 2 µM for the *wt*, which agrees with the observed changes in initial uptake rates. On 2-thiourea, A110G and Y437F show a moderate sensitivity at concentrations of 5 mM, while N275Q is already sensitive at concentrations of 2.5 mM (figure 3*a*). All three mutations result in *K_i_* values for 2-thiourea that are approximately twice as high as those of the *wt* and exceed the lowest concentrations used in plate tests (625 µM; not shown). As mentioned above, this may explain why phenotypic differences became visible for these mutants. Additionally, *K_i_* values suggest that Y437F results in an augmented apparent affinity for acetamide (102 ± 15 µM compared with 237 ± 14 µM in the *wt*; table 1), while A110G and N275Q result in reduced affinity for acetamide (495 ± 29 µM and 352 ± 26 µM, respectively, compared with 237 ± 14 µM in the *wt*; table 1). S446T does not affect the phenotype in growth tests nor does it change significantly the uptake kinetic constants.

Epifluorescence microscopy was used to investigate the subcellular localization of GFP-tagged UreA versions bearing all the above-mentioned mutations. With the exception of S446L, none of these mutations (Y106A/F, A110D/G, N275I/Q, Y437A/F or S446T) affect the membrane localization of UreA (electronic supplementary material, figure S3). In non-repressive conditions (proline as nitrogen source), the GFP signal is seen predominantly in the plasma membrane and the septae, and to a lesser extent in cytoplasmic organelles previously identified as endosomes and vacuoles [11]. S446L results in a completely different localization, with fluorescence accumulating in ring-shaped structures around the nucleus (figure 3*c*), a pattern typical of endoplasmic reticulum (ER)-resident proteins [41,42]. Fluorescence also accumulates in vacuoles. The perinuclear localization was confirmed with a strain carrying UreAS446L-GFP and a H1-mRFP fusion [43,44] (figure 3*c*). This localization suggests that changing S446 into an L may be detrimental for proper folding of UreA or for its trafficking towards the plasma membrane, causing retention in the ER. Western blots show a diminished amount of total UreA protein as compared with the *wt* (intact UreA–GFP + free GFP, the latter resulting from UreA–GFP turnover; figure 3*b*).

### 3.2. Directed mutagenesis of residues in the putative substrate pathway

**Figure 4.**
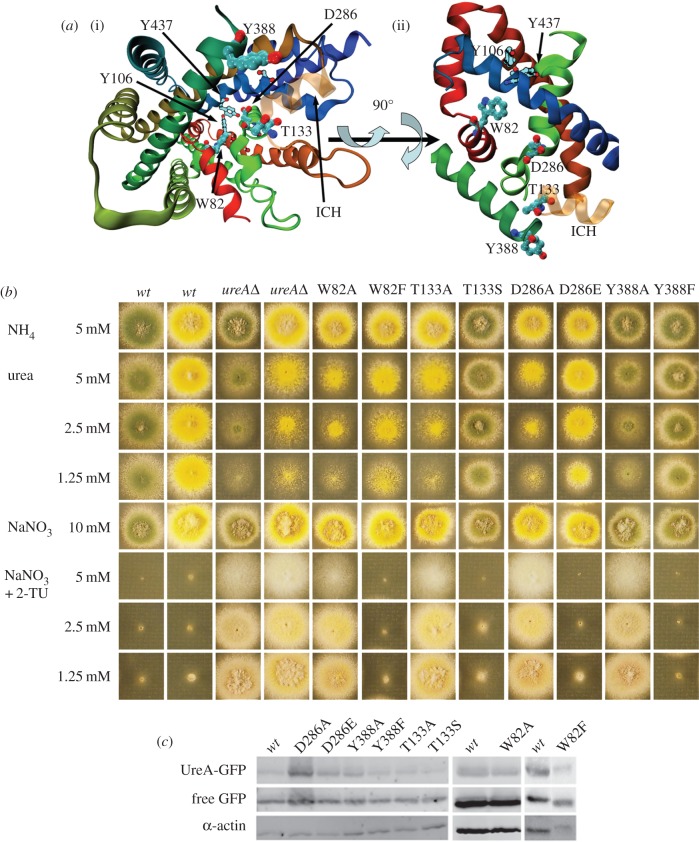
Analysis of strains bearing mutations in the putative intramolecular urea pathway. (*a*) Location of the mutated residues. (i) A perspective view into the inward-facing cavity. W82, T133, Y388 and D286 are shown from the cytoplasmic side. Y106 and Y437, which close the cavity, are shown for reference. The colouring of the cartoon is the same as in figure 2. The additional intracellular helix where T133 is located is shown as semitransparent in orange. (ii) A side view of the transporter showing the W82, T133, Y388 and D286 residues in the predicted urea path. For clarity, we show only the helixes where the residues chosen for directed mutagenesis are located. (*b*) Growth tests of strains bearing mutations in W82, T133, Y388 and D286 residues. Growth conditions were as described in figure 3*a*. *yA*^+^ and *yA2* *wt* strains were used as positive controls so that each mutant strain could be compared with the relevant one. A *ureA*Δ strain is shown as a negative control. (*c*) Western blot analysis on total protein extracts of UreA–GFP mutants probed with anti-GFP antibody. See legend of figure 3*b* for details.

We then proceeded to analyse in more detail the conservation of residues lining the putative pathway of urea through the transporter. We reasoned that residues involved in this function should (i) be conserved among the family of urea transporters, (ii) be solvent exposed in the structural model, and (iii) possess polar physicochemical properties to interact with the urea molecule. This analysis led to the identification of four amino acids: W82, T133, D286 and Y388 (figure 4*a*). W82 is situated in TMS2, T133 in the intracellular helix found between TMS3 and TMS4 (ICH3/4), D286 in TMS7, and Y388 at the end of TMS9 (electronic supplementary material, figure S1). Interestingly, the latter is among the amino acids identified as putatively involved in transporter functionality because of its conservation in functionally characterized UreA orthologues but not in its paralogues (see above).

These four residues were mutated to alanine and conservative changes were also introduced at these positions. Phenotypic analysis of mutant strains, apparent urea transport rates and uptake competition assays were carried out as mentioned above. In agreement with structural predictions, W82A, T133A, D286A and Y388A result in loss of function of UreA, because mutants carrying them are unable to grow on urea at concentrations of 0.625–2.5 mM and are resistant to 2-thiourea when present at the same concentration range (figure 4*b*). Accordingly, initial urea uptake rates are in all cases almost undetectable (less than 2%; table 1).

Mutants carrying the D286E and Y388F conservative changes grow normally on urea as sole nitrogen source and show normal sensitivity to 2-thiourea (figure 4*b*). Strains carrying these mutations show 100% apparent urea uptake rates and *K_m_*_/*i*_ affinity constants for urea, 2-thiourea, acetamide and guanidine that are not significantly different from those of the *wt* strain (table 1). T133S is phenotypically indistinguishable from the *wt* (figure 4*b*). Nevertheless, this mutation results in an initial urea uptake rate of approximately 70% of that of the *wt.* This may be explained by a reduced affinity for urea (*K_m_* 80% higher compared with the *wt*). *K_i_* for 2-thiourea and acetamide is also increased (approximately 30%) for these two substrates (table 1). Finally, mutant W82F behaves phenotypically on urea as a partial loss-of-function mutant. By contrast, no phenotypic differences are obvious on 2-thiourea compared with the *wt* (figure 4*b*). The apparent urea uptake capacity for this mutant is approximately 50% of that of the *wt*. However, its *K_m_* for urea is not significantly different to that of the *wt* (table 1), which means that even if the substrate could bind efficiently, the mechanism of translocation of urea across the membrane is somehow hindered by the W82F mutation. Moreover, *K_i_* values for other substrates are slightly increased, which points to a moderate role of this residue in substrate selectivity. Epifluorescence observations of this set of mutants show that in all of them UreA localizes normally to the membrane (electronic supplementary material, figure S3). The cognate mutations do not produce drastic differences in protein concentrations in total preparations (figure 4*c*), as demonstrated by Western blots.

### 3.3. Mutations obtained by random mutagenesis

**Figure 5.**
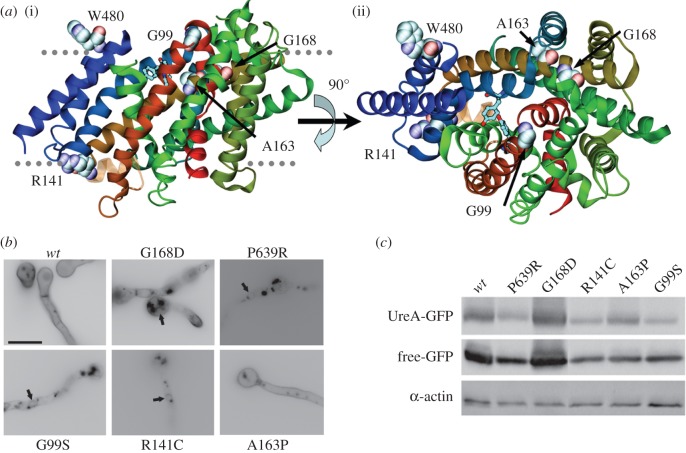
Analysis of mutants isolated by classical random mutagenesis. (*a*) Structural context of mutants isolated by classical random mutagenesis. (i) The UreA molecule is shown as helix-succession representation, coloured as in figure 2. The residues affected by the mutations are shown with a space-filling representation. For reference, Y106, A110, N275, Y437 and S446 are shown by stick representation. (ii) Same as (i), but seen from the extracellular side. Stick representations of Y106 and Y437 are included for reference. (*b*) Epifluorescence microscopy in greyscale inverted mode, showing *in vivo* subcellular expression of mutant UreA–GFP fusions. Arrows signal perinuclear ER membrane rings. (*c*) Western blot analysis on total protein extracts of UreA–GFP mutants probed with anti-GFP antibody. See legend of figure 3*b* for details.

As a complementary strategy, the isolation of *ureA* mutants by classical random mutagenesis (as described in Experimental procedures) and the characterization of these and previously isolated spontaneous mutants were carried out. Mutated residues are summarized in table 2 and shown in figure 5*a*.

**Table 2.** Summary of UreA mutations obtained by classical random mutagenesis. TMS, transmembrane segment; TMS2/TMS3, loop between TMS2 and TMS3; ICH3/4, intracellular helix between TMS3 and TMS4; C-ter, C-terminal region; M, localization to the membrane; ER, retention in the endoplasmic reticulum; n.d., not determined.

amino acid substitution	origin	location in protein	intracellular localization
G168D (*ureA*1)	[16]	TMS4	ER
P639R (*ureA9*05)	[11]	C-ter	ER
G99S	this study	TMS2/TMS3	ER
R141C	this study	ICH3/4	ER
A163P	this study	TMS4	M
W480Stop	this study	TMS12	n.d.
Q570Stop	this study	TMS13/TMS14	n.d.

Mutation G168D [11,16] is predicted by TMHMM [45] and our structural model to affect the last residue in TMS4, while mutation P639R [11] affects the first residue in the C-terminal region of the protein (figures 1 and 5*a*). This region is not included in our structural model. Mutants bearing G168D or P639R show only a residual growth on urea, similar to that of the *ureA*Δ strain [11]. ^14^C-urea transport assays agree with these results [11]. Epifluorescence microscopy observations of GFP fusions of these mutant proteins show the characteristic localization of ER-retained proteins, in ring-shaped structures around nuclei (figure 5*b*), whereas in the case of mutation G168D some fluorescence is also seen in the plasma membrane.

Five novel mutations were selected for further work, as described in Experimental procedures: G99S (predicted to be in the extracellular loop between TMS2 and TMS3), R141C (ICH3/4), A163P (in TMS4), W480 to a STOP codon (end of TMS12) and Q570 to a STOP codon (predicted to be in the extracellular loop between TMS13 and TMS14) (figures 1 and 5*a*). Mutants bearing any of these changes behave like the *ureA*Δ mutant in terms of growth phenotypes on urea and 2-thiourea, when incubated at 37°C (electronic supplementary material, figure S4) or 25°C (not shown). No differences were observed in terms of initial uptake rates with respect to the *ureA*Δ strain (not shown).

Epifluorescence microscopy showed that in the A163P mutant, UreA localization is indistinguishable from that of a *wt* strain, staining mainly the membrane and septae, while mutations G99S and R141C result in UreA retention in the ER (figure 5*b*). Fluorescence is also present in vacuoles in both mutants, a fact that could imply an ER-to-vacuole degradation route [46] or transient plasma membrane localization followed by degradation due to protein instability.

Western blots of total protein extracts (figure 5*c*) show that neither diminution of intact UreA–GFP levels nor any increase in degradation (as shown by the relative level of free GFP) occurs in these mutants. The increase in protein level observed for G168D could be due to an increase in the intrinsic stability of the mutant protein or to reduced vacuolar turnover due to ER retention [47].

### 3.4. Modelling of UreA in the outward-facing cavity conformation

**Figure 6.**
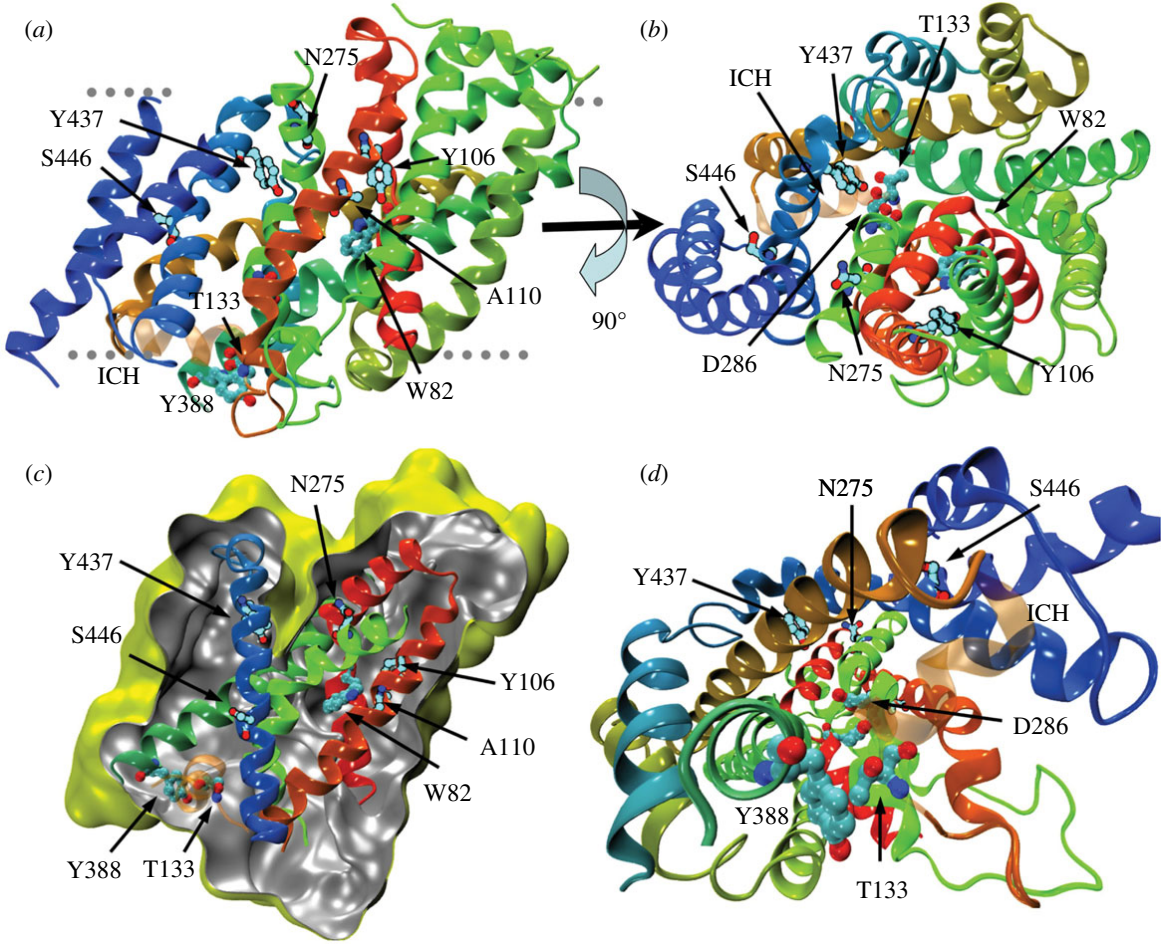
Molecular model of UreA in the outward-facing conformation. (*a*) Secondary structure elements and amino acids are coloured as in figure 2. The approximate position of the membrane is indicated by the grey dotted lines with the extracellular side on the upper part of the figure. (*b*) Same as in (*a*), rotated 90° along the membrane plane and seen from the extracellular side. (*c*) Solvent accessible surface calculated with a probe radius of 0.2 nm. The slice on the surface is taken perpendicular to the membrane to show the outward-facing cavity. External and internal sides of the surface are yellow and grey, respectively. For clarity, we show only the helixes where the residues chosen for directed mutagenesis are located. (*d*) Perspective view into the outward-facing cavity from the intracellular side (compare with figure 5*a*). The colour of the cartoon is the same as figure 2, and the intracellular helix where T133 is located is shown as semitransparent.

Results obtained for strains bearing mutations Y106F and W82F show that there is a reduction in the initial transport rate which does not correlate with *K_m_* and *K_i_* changes (table 1). This may suggest that urea can bind to the protein but the mutation somehow impairs the conformational rearrangements of the protein along the translocation process. To acquire a different structural perspective on the role of these residues, we undertook the construction of a model of UreA based on the structure of the Nucleobase-Cation-Symport-1 benzylhydantoin transporter from *M. liquefaciens* (Mhp1; PDB code 2JLN [29]). Although Mhp1 shows less amino acid sequence identity with UreA than vSGLT (see Experimental procedures), this model was constructed to obtain an insight into the possible role of the amino acids identified in the outward closed conformation. As it can be expected, the models constructed using structural templates in inward closed and outward closed conformations are significantly different. Indeed, the positions and interactions of most of the residues undergo substantial changes upon the conformational transition from the inward- to the outward-facing cavity (figure 6; electronic supplementary material, movie).

The large rearrangements undergone by Y106 and W82 comparing inward- and outward-facing conformations lead us to speculate that changing any of them into Phe may impair the conformational transition, without substantially affecting the binding of urea in the outward facing cavity. The different perspective provided by this structural model may also suggest a possible role for R141. In the conformation presenting the outward-facing cavity, R141 is in good position to form a salt bridge interaction with E123 (electronic supplementary material, figure S5), which is conserved only in AtDUR3 and OsDUR3 (figure 1). This salt bridge would be disrupted by the R141C mutation, causing a structural modification that may ultimately lead to the retention of UreA in the ER.

## 4. Discussion

In this work we present, based on a strategy combining site-directed and classical random mutagenesis with three-dimensional modelling, the first structure–function study of a protein of the group of urea/H^+^ transporters of fungi and plants, the *A. nidulans* UreA. Although the reliability of the modelling is limited by the relatively low amino acid identity with structurally characterized homologues, the models obtained allowed pinpointing specific amino acids, which, together with classical random mutagenesis, led to the identification of specific residues important for the function of UreA, some of which are crucially involved urea uptake by UreA.

We selected residues W82, Y106, A110, T133, N275, D286, Y388, Y437 and S446 for site-directed mutagenesis. Except for N275 and S446, these residues are predicted by both our three-dimensional models to be exposed to the cavity and thus would be able to establish contacts with the substrate. N275 and S446 are predicted to be in helixes that line the cavity, but are not exposed to it.

Interestingly, our model suggests that W82, Y106, A110, N275, D286, Y437 and S446 are located in TMSs 2, 3, 7 and 11. These helixes correspond to helixes 2, 3, 7 and 11 in vSGLT, where they are involved in substrate binding and recognition [30,48].

Our data indicate that the above-mentioned residues would be involved in urea transport at different steps of the substrate translocation process. With the exception of S446, non-conservative mutations of these residues do not alter the sorting of UreA mutant proteins towards the membrane; transport impairment must therefore be due to reduced binding or translocation of the substrate across the membrane. With the exception of D286E, Y388F and S446T, all conservative changes introduced in UreA affect urea transport. A reduction in initial transport rates correlates well with changes in *K_m_* and *K_i_*, except for strains carrying mutations W82F and Y106F, whose *K_m_*_/*i*_ values do not fully explain the impairment in uptake rates. It can then be speculated that these mutations must also affect other aspects of the transporter's function. Finally, changes in *K_m_* and *K_i_* for substrates and inhibitors resulting from mutations Y106F, A110G, T133S and Y437F suggest involvement of these residues in UreA substrate selectivity.

5-HIR transporters are characterized by the presence of filters or gates that determine the open or occluded states in the inward- and outward-facing conformations, which may have a role in substrate selectivity [37,40]. The location of residues Y106 and Y437, which, according to the model, are predicted to face each other in the inward closed conformation, could suggest a role of these two aromatic residues as a filter gate [37,49,50]. Moreover, these two residues lay in a position that almost superimposes with residues shown to be part of the outside gate in vSGLT. The fact that these two residues seem to play a role in substrate selectivity reinforces this idea. Our molecular model of the UreA in the inward-facing, closed conformation suggests that selective permeation may be regulated in a similar fashion to that deduced from the crystal structure of a bacterial channel-like urea transporter, dvUT [51]. In this case, urea would pass through a selectivity filter where a couple of facing Phe residues ‘sandwich’ the substrate, so that the aromatic rings stabilize the partial positive charge on the amide nitrogen by amide–PI stacking interactions.

Mutation S446L is unique among the site-directed mutations studied in this work, in the sense that it is the only one among the rationally designed mutations that abolishes the correct trafficking or insertion of the mutant protein into the plasma membrane, while the substitution by threonine maintains normal function. This suggests that a non-conservative change may lead to an aberrant folding of the mutant protein.

Classical random mutagenesis led to the isolation of mutants in TMS4, in the loops between TMS2 and TMS3, TMS3 and TMS4 and TMS4, and TMS5, and also at the beginning of the predicted C-terminal region of the protein. Except for mutation A163P, all other changes lead to the retention of the mutant protein in the ER. This suggests a relevant role of these residues in the establishment of proper folding of the protein or in its correct traffic to the membrane. It is worth noting that in support of a role in the determination of the basic transporter's structure, all the residues identified by classical random mutagenesis show a high degree of conservation not only among UreA orthologues but also, for a number of them, among SSS members. Residues G99 and R141 are conserved in UreA orthologues and in all members of the SSS family characterized so far (figure 1; electronic supplementary material, figure S2), while G168 is partially conserved in characterized SSS members, where a serine is found in the same position in *S. cerevisiae* DUR3 and a proline in NIS (human sodium-iodide symporter; electronic supplementary material, figure S2). P639 is conserved in all UreA orthologues, but not in SSS members. One possible hypothesis is that the mutation of this residue abolishes a kink at the carboxy terminus of TMS15, which may be important for the correct orientation of the C-terminal region of UreA. C- and N-terminal tails are characteristic of eukaryotic transporters bearing 5-HIR structures [27,28,47,52], while in bacterial transporters of this family these regions are not present. A163 is the less conserved residue identified by random mutagenesis (figures 1; electronic supplementary material, S2); it is substituted by a serine in ScDUR3, PiDur3 and CaDur3, and is not conserved at all in non-UreA homologues of the SSS. It could be speculated that a proline substitution would introduce a kink in helix TMS4.

The work presented here underlines the opportunity to combine modelling with site-directed and random mutagenesis analysis. While directed mutagenesis suggested the important role of residues for substrate binding and translocation, random mutagenesis identified residues involved in protein translocation to the membrane and the possible role of a salt bridge to stabilize the conformation of the protein.

Finally, our work could not provide, in the absence of other specific information, any insight on the amino acids or regions of UreA involved in proton symport. In previous work [11], we performed inhibition experiments in the presence of the H^+^-uncoupler CCCP and the H^+^-ATPase inhibitor DCCD, which completely abolish urea uptake by UreA. These experiments, despite being indirect, show that, like most fungal transporters, UreA operates as an H^+^/symporter. However, there is no way of distinguishing mutations resulting in an impairment of H^+^/symport from other loss-of-function mutations, so no conclusions can be drawn from inhibition experiments of mutants. In this work, all mutants that conserved some activity, when tested in the presence of CCCP, showed abolished uptake similarly to the *wt*, therefore these results were not shown.

## 5. Experimental procedures

### 5.1. Strains, media and transformation procedures

Standard complete and minimal media (MM) for *A. nidulans* were employed [53,54] (http://www.fgsc.net). Supplements were added when necessary at standard concentrations (http://www.gla.ac.uk/ibls/molgen/aspergillus/supplement.html). *Aspergillus nidulans* strains used in this study are listed in the electronic supplementary material, table S1. Gene symbols are defined in http://www.gla.ac.uk/ibls/molgen/aspergillus/loci.html. Urea (0.625–5 mM), NaNO_3_ (10 mM), ammonium L(+)-tartrate (5–10 mM) or proline (5 mM) were used as sole N-sources. 2-thiourea was used in concentrations of 0.625–5 mM. *Aspergillus nidulans* transformation was carried out as in [55].

### 5.2. Generation of *ureA* mutants

#### 5.2.1. Site-directed mutagenesis

Site-directed mutagenesis on the *ureA*::*gfp* gene was performed by the Fusion-PCR technique [55] using KAPA HiFi DNA polymerase (KAPA Biosystems) with primers Ure5-F, Ure3-R and complementary ones carrying the desired substitution (electronic supplementary material, table S2). DNA extracted from a *ureA*::*gfp*::*AFpyrG* strain (MVD 10A) was used as template. A resulting 7 kb fusion product was amplified with nested primers Ure5-N and Ure3-N and purified with the GeneJET Gel Extraction Kit (Thermo Scientific). The resulting construct was transformed in a *ureA*Δ::*riboB pyrG89 pyroA4 riboB2 nkuA*Δ::*argB veA1* strain (MVD 13A). Protoplasts were plated on selective (containing riboflavin, but not uridine and uracil) 1 M sucrose MM and incubated at 37°C. Transformants were purified on selective MM and its ability to grow both in different concentrations of urea and 2-thiourea was tested. Southern blots were performed in order to check the integration to the *ureA* locus and to assess the number of construction copies integrated in the genome. Probes were labelled with the DIG DNA labelling and detection kit (Roche Applied Science). Sequencing of the resulting mutants was performed at Macrogen, Inc. (Seoul, Korea).

#### 5.2.2. Classical random mutagenesis

Classical random mutagenesis was performed by incubating a 10 ml spore suspension from strain MVD 10A with 20 µl of 4-nitroquinoline 1-oxide (1 µg ml^−1^) for 1 h at 37°C. Selection of 2-thiourea-resistant strains was performed on medium containing 1.25 mM 2-thiourea using NaNO_3_ (10 mM) as a nitrogen source. Five mutants were selected for further analysis. Crossover with an *ureA*1 strain (MVD 100) was performed in order to verify that the produced mutation was in the *ureA* locus. Sequencing of *ureA* mutants was performed at Macrogen, Inc.

### 5.3. Radiolabelled urea uptake measurements

[^14^C]-urea uptake in MM was assayed in germinating conidiospores of *A. nidulans* at 37°C, pH 6.8, as previously described [11]. Initial velocities were measured at a time point of incubation where velocity increases linearly (2 min), using a concentration of 1.0 µM [^14^C]-urea, and are expressed as % of the *wt* in pmoles min^−1^/10^7^ conidiospores. *K_m_*_/*i*_ values were obtained directly by performing and analysing uptakes (Prism 3.02; Graph Pad Software, Inc.), using labelled urea at 0.5–50 μM, or in the presence of 1.0 µM [^14^C]-urea and of various concentrations (0.5–3000 μM) of non-labelled substrates. *K_i_* values were calculated from the Cheng and Prusoff equation *K_i_* = IC_50_/(1 + (*L*/*K_m_*), in which *L* is the permeant concentration. Transport assays were carried out in at least three independent experiments, with three replicates for each concentration or time point. Background uptake values were corrected by subtracting values measured in the deleted mutants (UreAΔ). Standard deviation was less than 20%. Radiolabelled [^14^C]-urea (55.0 mCi mmol^−1^) was purchased from Moravek Biochemicals, Brea, CA, USA.

### 5.4. Protein extraction and Western blot

Total protein of 200 mg of grinded mycelia was extracted as described by Apostolaki *et al.* [56] and protein concentration was determined by the Bradford assay. Total proteins (50 µg) were separated by SDS-PAGE (10% (w/v) polyacrylamide gel) and electroblotted (OmniPAGE Electroblotting Units, Cleaver Scientific Ltd) onto a nitrocellulose membrane (0.45 µm, Thermo Scientific). Membrane was then incubated in stripping buffer (10 mM Tris–HCl pH 6.8, 2% SDS and 100 mM β-mercaptoethanol) for 15 min at 55°C (as described by Kaur & Bachhawat [57]). Membrane was then treated with 5% non-fat dry milk, and immunodetection was done with a primary mouse anti-GFP monoclonal antibody (Roche Applied Science), or a mouse anti-actin monoclonal (C4) antibody (MP Biomedicals) and a secondary sheep anti-mouse IgG HRP-linked antibody (GE Healthcare). Blots were developed using the Amersham ECL Western blotting detection reagents and analysis system (GE Healthcare), and images were acquired using the GeneSys software from the GBoxChemi XT4 System (Syngene).

### 5.5. Epifluorescence microscopy

Samples for fluorescence microscopy were prepared as described previously [58]. In brief, samples were incubated directly on sterile coverslips protected from light in liquid MM with proline (5 mM) as nitrogen source and appropriate supplements, at 25°C for 14–16 h. For epifluorescence microscopy, samples were observed in an Olympus inverted microscope CKX31 with a U-MNIBA3 (for GFP) or U-MWIG3 (for RFP) filter, photographed with a Hamamatsu Orca Er camera and processed using Image Pro v. 6.0 software. For confocal microscopy, samples were observed in a Leica inverted microscope DMI6000CS with an I3 (for GFP) or N2.1 (for RFP) filter. Images were acquired and processed using LASAF v. 2.20 software. The resulting images were processed by Adobe Photoshop software. Microscopy facilities belonging to the Cellular Biology Platform, Institut Pasteur de Montevideo, were used.

### 5.6. Bioinformatics and modelling of UreA

Sequences were obtained from the Aspergillus Comparative Database, Broad Institute (http://www.broad.mit.edu/annotation/fungi/aspergillus), the Saccharomyces genome database (http://www.yeastgenome.org), the Candida Genome Database (http://www.candidagenome.org), The Arabidopsis Information Resource (http://www.arabidopsis.org), the *Oryza sativa* genome database (http://www.plantgdb.org/OsGDB) and from the National Center for Biotechnology Information (http://www.ncbi.nlm.nih.gov). PiDUR3 sequence was kindly provided by Morel *et al.* [10]. Multiple sequence alignments were carried out in MEGA v. 5.0 software [59] with Muscle [60]. Homologues of UreA with known structure were identified by performing a remote homology search on the Protein Data Bank (http://www.pdb.org) using Hidden Markov model profiles with Hhpred [61]. The best structural template in the closed inward-facing conformation corresponds to the structure of vSGLT of *V. parahaemolyticus* [30] with a sequence identity of 18%, and *E*- and *p*-values of 3.77 × 10^−65^ and 1.2 × 10^−69^, respectively. The best structural template in the closed outward-facing conformation corresponds to the structure of Mhp1 from *M. liquefaciens* [29] with a sequence identity of 14%, and *E*- and *p*-values of 5.6 × 10^−09^ and 1.8 × 10^−13^, respectively. Molecular models on both conformations comprising amino acids 64–525, spanning TMS 2–13, were built using those templates and the sequence alignments obtained from HHpred with MODELLER 9.12 [62]. In both cases, 1000 models were generated, from which the best 10 were selected on the base of the Modeller's Objective Function. The stereochemical properties of the remaining models were assessed using Procheck [63]. The models with highest number of amino acids in most favoured regions and higher overall G-factors were selected as representatives of the inward- and outward-facing conformations (electronic supplementary material, table S3). In both cases, they are comparable or better than expected for medium-quality X-ray structure.

## Supplementary Material

Supplementary Material Figures and Tables

## References

[1] McDonald MD Smith CP Walsh PJ 2006 The physiology and evolution of urea transport in fishes. J. Membr. Biol. 212, 93–107. (doi:10.1007/s00232-006-0869-5) 17264987 10.1007/s00232-006-0869-5

[2] Witte CP 2011 Urea metabolism in plants. Plant Sci. 180, 431–438. (doi:10.1016/j.plantsci.2010.11.010) 21421389 10.1016/j.plantsci.2010.11.010

[3] Wagemaker MJ Welboren W van der Drift C Jetten MS Van Griensven LJ Op den Camp HJ 2005 The ornithine cycle enzyme arginase from *Agaricus bisporus* and its role in urea accumulation in fruit bodies. Biochim. Biophys. Acta 1681, 107–115. (doi:10.1016/j.bbaexp.2004.10.007) 15627502 10.1016/j.bbaexp.2004.10.007

[4] Smith CP 2009 Mammalian urea transporters. Exp. Physiol. 94, 180–185. (doi:10.1113/expphysiol.2008.043042) 19028811 10.1113/expphysiol.2008.043042

[5] Davis RH 1970 Sources of urea in *Neurospora*. Biochim. Biophys. Acta 215, 412–414. (doi:10.1016/0304-4165(70)90042-5) 5503395 10.1016/0304-4165(70)90042-5

[6] Satoh Y 1980 Production of urea by bacterial decomposition of organic matter including phytoplankton. Int. Revue der gesamten Hydrobiol. Hydrographie 65, 295–301. (doi:10.1002/iroh.19800650216)

[7] Glibert PM Harrison J Heil C Seitzinger S 2006 Escalating worldwide use of urea: a global change contributing to coastal eutrophication. Biogeochemistry 77, 441–463. (doi:10.1007/s10533-005-3070-5)

[8] Saier MH Jr Reddy VS Tamang DG Vastermark A 2014 The transporter classification database. Nucleic Acids Res. 42, D251–D258. (doi:10.1093/nar/gkt1097) 24225317 10.1093/nar/gkt1097 PMC3964967

[9] ElBerry HM Majumdar ML Cunningham TS Sumrada RA Cooper TG 1993 Regulation of the urea active transporter gene (DUR3) in *Saccharomyces cerevisiae*. J. Bacteriol. 175, 4688–4698. 8335627 10.1128/jb.175.15.4688-4698.1993 PMC204920

[10] Morel M Jacob C Fitz M Wipf D Chalot M Brun A 2008 Characterization and regulation of PiDur3, a permease involved in the acquisition of urea by the ectomycorrhizal fungus *Paxillus involutus*. Fungal Genet. Biol. 45, 912–921. (doi:10.1016/j.fgb.2008.01.002) 18313954 10.1016/j.fgb.2008.01.002

[11] Abreu C Sanguinetti M Amillis S Ramon A 2010 UreA, the major urea/H^+^ symporter in *Aspergillus nidulans*. Fungal Genet. Biol. 47, 1023–1033. (doi:10.1016/j.fgb.2010.07.004) 20633690 10.1016/j.fgb.2010.07.004

[12] Navarathna DH Das A Morschhauser J Nickerson KW Roberts DD 2011 Dur3 is the major urea transporter in *Candida albicans* and is co-regulated with the urea amidolyase Dur1,2. Microbiology 157, 270–279. (doi:10.1099/mic.0.045005-0) 20884691 10.1099/mic.0.045005-0 PMC3069533

[13] Liu LH Ludewig U Frommer WB von Wiren N 2003 AtDUR3 encodes a new type of high-affinity urea/H^+^ symporter in *Arabidopsis*. Plant Cell 15, 790–800. (doi:10.1105/tpc.007120) 12615950 10.1105/tpc.007120 PMC150031

[14] Merigout P Lelandais M Bitton F Renou JP Briand X Meyer C Daniel-Vedele F 2008 Physiological and transcriptomic aspects of urea uptake and assimilation in *Arabidopsis* plants. Plant Physiol. 147, 1225–1238. (doi:10.1104/pp.108.119339) 18508958 10.1104/pp.108.119339 PMC2442537

[15] Wang WH 2012 Rice DUR3 mediates high-affinity urea transport and plays an effective role in improvement of urea acquisition and utilization when expressed in *Arabidopsis*. New Phytol. 193, 432–444. (doi:10.1111/j.1469-8137.2011.03929.x) 22010949 10.1111/j.1469-8137.2011.03929.x

[16] Pateman JA Dunn E Mackay EM 1982 Urea and thiourea transport in *Aspergillus nidulans*. Biochem. Genet. 20, 777–790. (doi:10.1007/BF00483973) 6814421 10.1007/BF00483973

[17] Vadyvaloo V Smirnova IN Kasho VN Kaback HR 2006 Conservation of residues involved in sugar/H(+) symport by the sucrose permease of *Escherichia coli* relative to lactose permease. J. Mol. Biol. 358, 1051–1059. (doi:10.1016/j.jmb.2006.02.050) 16574149 10.1016/j.jmb.2006.02.050 PMC2786776

[18] Kaback HR Dunten R Frillingos S Venkatesan P Kwaw I Zhang W Ermolova N 2007 Site-directed alkylation and the alternating access model for LacY. Proc. Natl Acad. Sci. USA 104, 491–494. (doi:10.1073/pnas.0609968104) 17172438 10.1073/pnas.0609968104 PMC1766412

[19] Tavoulari S Frillingos S 2008 Substrate selectivity of the melibiose permease (MelY) from *Enterobacter cloacae*. J. Mol. Biol. 376, 681–693. (doi:10.1016/j.jmb.2007.12.015) 18177889 10.1016/j.jmb.2007.12.015

[20] Yousef MS Guan L 2009 A 3D structure model of the melibiose permease of *Escherichia coli* represents a distinctive fold for Na^+^ symporters. Proc. Natl Acad. Sci. USA 106, 15 291–15 296. (doi:10.1073/pnas.0905516106) 10.1073/pnas.0905516106 PMC2729278 19706416

[21] Sugihara J Smirnova I Kasho V Kaback HR 2011 Sugar recognition by CscB and LacY. Biochemistry 50, 11 009–11 014. (doi:10.1021/bi201592y) 10.1021/bi201592y PMC3249425 22106930

[22] Ethayathulla AS Yousef MS Amin A Leblanc G Kaback HR Guan L 2014 Structure-based mechanism for Na^+^/melibiose symport by MelB. Nat. Commun. 5, 3009 (doi:10.1038/ncomms4009) 24389923 10.1038/ncomms4009 PMC4026327

[23] Zhang YW Rudnick G 2006 The cytoplasmic substrate permeation pathway of serotonin transporter. J. Biol. Chem. 281, 36 213–36 220. (doi:10.1074/jbc.M605468200) 17008313 10.1074/jbc.M605468200

[24] Zomot E Bendahan A Quick M Zhao Y Javitch JA Kanner BI 2007 Mechanism of chloride interaction with neurotransmitter : sodium symporters. Nature 449, 726–730. (doi:10.1038/nature06133) 17704762 10.1038/nature06133

[25] Beuming T 2008 The binding sites for cocaine and dopamine in the dopamine transporter overlap. Nat. Neurosci. 11, 780–789. (doi:10.1038/nn.2146) 18568020 10.1038/nn.2146 PMC2692229

[26] Forrest LR Zhang YW Jacobs MT Gesmonde J Xie L Honig BH Rudnick G 2008 Mechanism for alternating access in neurotransmitter transporters. Proc. Natl Acad. Sci. USA 105, 10 338–10 343. (doi:10.1073/pnas.0804659105) 10.1073/pnas.0804659105 PMC2480614 18647834

[27] Kosti V Lambrinidis G Myrianthopoulos V Diallinas G Mikros E 2012 Identification of the substrate recognition and transport pathway in a eukaryotic member of the nucleobase-ascorbate transporter (NAT) family. PLoS ONE 7, e41939 (doi:10.1371/journal.pone.0041939) 22848666 10.1371/journal.pone.0041939 PMC3405029

[28] Amillis S Kosti V Pantazopoulou A Mikros E Diallinas G 2011 Mutational analysis and modeling reveal functionally critical residues in transmembrane segments 1 and 3 of the UapA transporter. J. Mol. Biol. 411, 567–580. (doi:10.1016/j.jmb.2011.06.024) 21722649 10.1016/j.jmb.2011.06.024

[29] Weyand S 2008 Structure and molecular mechanism of a nucleobase-cation-symport-1 family transporter. Science 322, 709–713. (doi:10.1126/science.1164440) 18927357 10.1126/science.1164440 PMC2885439

[30] Faham S Watanabe A Besserer GM Cascio D Specht A Hirayama BA Wright EM Abramson J 2008 The crystal structure of a sodium galactose transporter reveals mechanistic insights into Na^+^/sugar symport. Science 321, 810–814. (doi:10.1126/science.1160406) 18599740 10.1126/science.1160406 PMC3654663

[31] Singh SK Piscitelli CL Yamashita A Gouaux E 2008 A competitive inhibitor traps LeuT in an open-to-out conformation. Science 322, 1655–1661. (doi:10.1126/science.1166777) 19074341 10.1126/science.1166777 PMC2832577

[32] Krishnamurthy H Gouaux E 2012 X-ray structures of LeuT in substrate-free outward-open and apo inward-open states. Nature 481, 469–474. (doi:10.1038/nature10737) 22230955 10.1038/nature10737 PMC3306218

[33] Yamashita A Singh SK Kawate T Jin Y Gouaux E 2005 Crystal structure of a bacterial homologue of Na^+^/Cl^−^-dependent neurotransmitter transporters. Nature 437, 215–223. (doi:10.1038/nature03978) 16041361 10.1038/nature03978

[34] Suzuki S Henderson PJ 2006 The hydantoin transport protein from *Microbacterium liquefaciens*. J. Bacteriol. 188, 3329–3336. (doi:10.1128/JB.188.9.3329-3336.2006) 16621827 10.1128/JB.188.9.3329-3336.2006 PMC1447452

[35] Ressl S Terwisscha van Scheltinga AC Vonrhein C Ott V Ziegler C 2009 Molecular basis of transport and regulation in the Na^+^/betaine symporter BetP. Nature 458, 47–52. (doi:10.1038/nature07819) 19262666 10.1038/nature07819

[36] Perez C Koshy C Yildiz O Ziegler C 2012 Alternating-access mechanism in conformationally asymmetric trimers of the betaine transporter BetP. Nature 490, 126–130. (doi:10.1038/nature11403) 22940865 10.1038/nature11403

[37] Shi Y 2013 Common folds and transport mechanisms of secondary active transporters. Annu Rev Biophys 42, 51–72. (doi:10.1146/annurev-biophys-083012-130429) 23654302 10.1146/annurev-biophys-083012-130429

[38] Jeschke G 2013 A comparative study of structures and structural transitions of secondary transporters with the LeuT fold. Eur. Biophys. J. 42, 181–197. (doi:10.1007/s00249-012-0802-z) 22552869 10.1007/s00249-012-0802-z PMC3578728

[39] Shimamura T 2010 Molecular basis of alternating access membrane transport by the sodium-hydantoin transporter Mhp1. Science 328, 470–473. (doi:10.1126/science.1186303) 20413494 10.1126/science.1186303 PMC2885435

[40] Diallinas G 2008 Biochemistry: an almost-complete movie. Science 322, 1644–1645. (doi:10.1126/science.1168107) 19074336 10.1126/science.1168107

[41] Erpapazoglou Z Kafasla P Sophianopoulou V 2006 The product of the SHR3 orthologue of *Aspergillus nidulans* has restricted range of amino acid transporter targets. Fungal Genet. Biol. 43, 222–233. (doi:10.1016/j.fgb.2005.11.006) 16531082 10.1016/j.fgb.2005.11.006

[42] Leung J Cameron AD Diallinas G Byrne B 2013 Stabilizing the heterologously expressed uric acid-xanthine transporter UapA from the lower eukaryote *Aspergillus nidulans*. Mol. Membr. Boil. 30, 32–42. (doi:10.3109/09687688.2012.690572) 10.3109/09687688.2012.690572 22694048

[43] Campbell RE Tour O Palmer AE Steinbach PA Baird GS Zacharias DA Tsien RY 2002 A monomeric red fluorescent protein. Proc. Natl Acad. Sci. USA 99, 7877–7882. (doi:10.1073/pnas.082243699) 12060735 10.1073/pnas.082243699 PMC122988

[44] Toews MW Warmbold J Konzack S Rischitor P Veith D Vienken K Vinuesa C Wei H Fischer R 2004 Establishment of mRFP1 as a fluorescent marker in *Aspergillus nidulans* and construction of expression vectors for high-throughput protein tagging using recombination *in vitro* (GATEWAY). Curr. Genet. 45, 383–389. (doi:10.1007/s00294-004-0495-7) 15071756 10.1007/s00294-004-0495-7

[45] Krogh A Larsson B von Heijne G Sonnhammer EL 2001 Predicting transmembrane protein topology with a hidden Markov model: application to complete genomes. J. Mol. Biol. 305, 567–580. (doi:10.1006/jmbi.2000.4315) 11152613 10.1006/jmbi.2000.4315

[46] Houck SA Ren HY Madden VJ Bonner JN Conlin MP Janovick JA Conn PM Cyr DM 2014 Quality control autophagy degrades soluble ERAD-resistant conformers of the misfolded membrane protein GnRHR. Mol. Cell 1, 166–179. (doi:10.1016/j.molcel.2014.02.025) 24685158 10.1016/j.molcel.2014.02.025 PMC4070183

[47] Krypotou E Kosti V Amillis S Myrianthopoulos V Mikros E Diallinas G 2012 Modeling, substrate docking, and mutational analysis identify residues essential for the function and specificity of a eukaryotic purine-cytosine NCS1 transporter. J. Biol. Chem. 287, 36 792–36 803. (doi:10.1074/jbc.M112.400382) 10.1074/jbc.M112.400382 PMC3481282 22969088

[48] Watanabe A Choe S Chaptal V Rosenberg JM Wright EM Grabe M Abramson J 2010 The mechanism of sodium and substrate release from the binding pocket of vSGLT. Nature 468, 988–991. (doi:10.1038/nature09580) 21131949 10.1038/nature09580 PMC3736980

[49] Smirnova I Kasho V Kaback HR 2011 Lactose permease and the alternating access mechanism. Biochemistry 50, 9684–9693. (doi:10.1021/bi2014294) 21995338 10.1021/bi2014294 PMC3210931

[50] Diallinas G Gournas C 2008 Structure–function relationships in the nucleobase-ascorbate transporter (NAT) family: lessons from model microbial genetic systems. Channels 2, 363–372. (doi:10.4161/chan.2.5.6902) 18981714 10.4161/chan.2.5.6902

[51] Levin EJ Quick M Zhou M 2009 Crystal structure of a bacterial homologue of the kidney urea transporter. Nature 462, 757–761. (doi:10.1038/nature08558) 19865084 10.1038/nature08558 PMC2871279

[52] Papageorgiou I Gournas C Vlanti A Amillis S Pantazopoulou A Diallinas G 2008 Specific interdomain synergy in the UapA transporter determines its unique specificity for uric acid among NAT carriers. J. Mol. Biol. 382, 1121–1135. (doi:10.1016/j.jmb.2008.08.005) 18718842 10.1016/j.jmb.2008.08.005

[53] Cove DJ 1966 The induction and repression of nitrate reductase in the fungus *Aspergillus nidulans*. Biochim. Biophys. Acta 113, 51–56. (doi:10.1016/S0926-6593(66)80120-0) 5940632 10.1016/s0926-6593(66)80120-0

[54] Scazzocchio C Arst HN Jr 1978 The nature of an initiator constitutive mutation in *Aspergillus nidulans*. Nature 274, 177–179. (doi:10.1038/274177a0) 351427 10.1038/274177a0

[55] Szewczyk E Nayak T Oakley CE Edgerton H Xiong Y Taheri-Talesh N Osmani SA Oakley BR 2006 Fusion PCR and gene targeting in *Aspergillus nidulans*. Nat. Protoc. 1, 3111–3120. (doi:10.1038/nprot.2006.405) 17406574 10.1038/nprot.2006.405

[56] Apostolaki A Harispe L Calcagno-Pizarelli AM Vangelatos I Sophianopoulou V Arst HN Jr Penalva MA Amillis S Scazzocchio C 2012 *Aspergillus nidulans* CkiA is an essential casein kinase I required for delivery of amino acid transporters to the plasma membrane. Mol. Microbiol. 84, 530–549. (doi:10.1111/j.1365-2958.2012.08042.x) 22489878 10.1111/j.1365-2958.2012.08042.x PMC3491690

[57] Kaur J Bachhawat AK 2009 A modified Western blot protocol for enhanced sensitivity in the detection of a membrane protein. Anal. Biochem. 384, 348–349. (doi:10.1016/j.ab.2008.10.005) 18952039 10.1016/j.ab.2008.10.005

[58] Valdez-Taubas J Harispe L Scazzocchio C Gorfinkiel L Rosa AL 2004 Ammonium-induced internalisation of UapC, the general purine permease from *Aspergillus nidulans*. Fungal Genet. Biol. 41, 42–51. (doi:10.1016/j.fgb.2003.09.003) 14643258 10.1016/j.fgb.2003.09.003

[59] Tamura K Peterson D Peterson N Stecher G Nei M Kumar S 2011 MEGA5: molecular evolutionary genetics analysis using maximum likelihood, evolutionary distance, and maximum parsimony methods. Mol. Biol. Evol. 28, 2731–2739. (doi:10.1093/molbev/msr121) 21546353 10.1093/molbev/msr121 PMC3203626

[60] Edgar RC 2004 MUSCLE: a multiple sequence alignment method with reduced time and space complexity. BMC Bioinform. 5, 113 (doi:10.1186/1471-2105-5-113) 10.1186/1471-2105-5-113 PMC517706 15318951

[61] Soding J Biegert A Lupas AN 2005 The HHpred interactive server for protein homology detection and structure prediction. Nucleic Acids Res. 33, W244–W248. (doi:10.1093/nar/gki408) 15980461 10.1093/nar/gki408 PMC1160169

[62] Sali A Potterton L Yuan F van Vlijmen H Karplus M 1995 Evaluation of comparative protein modeling by MODELLER. Proteins 23, 318–326. (doi:10.1002/prot.340230306) 8710825 10.1002/prot.340230306

[63] Laskowski RA Rullmannn JA MacArthur MW Kaptein R Thornton JM 1996 AQUA and PROCHECK-NMR: programs for checking the quality of protein structures solved by NMR. J. Biomol. NMR 8, 477–486. (doi:10.1007/BF00228148) 9008363 10.1007/BF00228148

