# Supplementary material for: Modelling and mutational analysis of Aspergillus nidulans UreA, a member of the subfamily of urea/H+ transporters in fungi and plants
Source: Open Biol. 2014 Jun 25;4(6):140070. doi: 10.1098/rsob.140070 (PMC4077062; doi:10.1098/rsob.140070)
Supplement: Supplementary Material Figures and Tables [file rsob140070supp1.pdf]

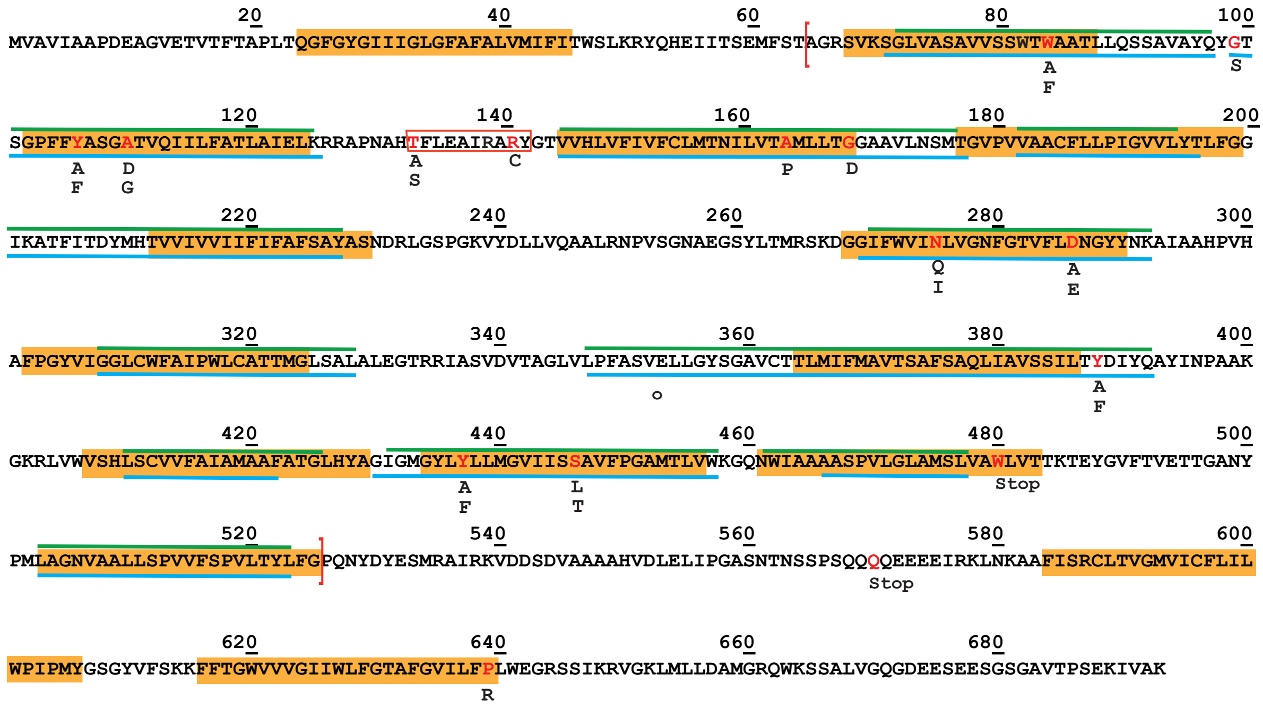

Figure S1. Sequence and structural prediction of UreA. The primary sequence corresponding to Gene Bank GI: 67516273 is shown along with transmembrane helix predictions (orange shading). Secondary structure elements resulting from homology modelling based on the X-ray structures of vSGLT (Protein Data Bank ID 3DH4) or Mhp1 (Protein Data Bank ID 2X79) are indicated by blue and green lines below and above the sequence, respectively. The red rectangle shows the position of an intracellular helix (ICH3/4), where T133 and R141 are located. The red brackets indicate the sequence included in the structural models. Amino acids mutated in this work are shown in red and substitutions are signalled below each residue.

```
UreA   62 FSTAGRSVKSGLVASAVVSSWTWAATLLQSSAVAYQYGTSGPFFYASGATVQIILFATLAIELKRRAPN---AHTFLEAIRA
ScDUR3 48 FTTAGRSVKTGLVAAAVVSSWIWCSTLLTSSTKEYADGIFGGYAYAAGACFQIIAFAILAIKTKQMAPN---AHTYLELVRT
AtDUR3 73 FNTAGRNVKTGLIASVIVSQWTWAATILQSSNVAWQYGVSGPFWYASGATIQVLLFGVMAIEIKRKAPN---AHTVCEIVKA
PiDUR3 47 FTSASRSVKPGLIASGIVSAWTWAATLLQSSAVAYRYGISGPWWYAAGATVQVLLFAQLAAKLKLNAPY---AHTWLEIVAT
CaDUR3 48 FATAGRSVKTGLIAACIVSSWTWSATLNGSTSQVYNNGVCGSIFYAMGATVQITLFACLAIKARERAPA---AHTYLEIVKG
OsDUR3 78 FNTAGRSVKTGLIASVIVSQWTWAATILQSSNVAWQYGVSGPFWYASGATIQVLLFGVMAIEIKRKAPN---AHTVCEIVRA
PutP   35 YILGGRSLGPFVTALSAGASDMSGWLLMGLPGAVFLSGISESWIAIGLTLGAWINWKLVAGRLRVHTEYNNNALTLPDYFTG
hSGLT  58 FFLAGRSMVWWPIGASLFASNIGSGHFVGLAGTGAASGIAIGGFEWNALVLVVVLGWLFVPIYIKAGVV-----TMPEYLRK
NIS    47 FFTGGRRLAALPVGLSLSASFMSAVQVLGVPSEAYRYGLKFLWMCLGQLLNSVLTALLFMPVFYRLGLT-----STYEYLEM
vSGLT  44 YFLAGKSLPWWAVGASLIAANISAEQFIGMSGSGYSIGLAIASYEWMSAITLIIVGKYFLPIFIEKGIY-----TIPEFVEK

UreA   141 RYG-TVVHLVFIVFCLMTNILVTAMLLT---GGAAVLNSMTGVPVVAACFLLPIGVVLYTLFGGIKATFITDYMHTVVIV
ScDUR3 127 RYG-KIGHGCYLFYAIATNILVTSMLLT-- SGSAVFSDLTGMNTIASCFLLPVGVVVYTLFGGIKATFLTDYMHTCVII
AtDUR3 152 RWG-TATHIVFLVFCLATNVVVTAMLLL---GGSAVVNALTGVNLYAASFLIPLGVVVYTLAGGLKATFLASYVHSVIVH
PiDUR3 126 RWG-TVAHLIFMFFGLATNLIVSSMLVL---GGSATVTSLTGMSTIAACFLIPLGVAIYVVVGGMRSTLLCDYTHTTVLF
CaDUR3 127 RYG-TTTHFVYIFWGIVTNILVTSMLLS---GGSATVNDLTGMNIVAAILLLPLGVVIYTLFGGIKATFLTDYAHTVVLI
OsDUR3 157 RWG-TPAHLVFLTFCLLTNVIVTAMLLL---GGSAVVNALTGVNVYAASFLIPLGVVVYTLAGGLKATFLASYIHSVVVH
PutP   117 RFE-DKSRILRIISALVILLFFTIYCASGIVAGARLFESTFGMSYETALWAGAAATILYTFIGGFLAVSWTDTVQASLMI
hSGLT  135 RFGGQRIQVYLSLLSLLLYIFTKISADIFS--GAIFINLALGLNLYLAIFLLLAITALYTITGGLAAVIYTDTLQTVIML
NIS    124 RFS-RAVRLCGTLQYIVATMLYTGIVIY---APALILNQVTGLDIWASLLSTGIICTFYTAVGGMKAVVWTDVFQVVVML
vSGLT  121 RFN-KKLKTILAVFWISLYIFVNLTSVLY--LGGLALETILGIPLMYSILGLALFALVYSIYGGLSAVVWTDVIQVFFLV
          . .  .  .  .  .  .  .  .  .  .
          . .  .  .  .  .  .  .  .  .  .

UreA   423 ATGLHYAGIGMG-YLYLLMGVIISSAVFPGAMTLVWKGQNWIAAAASPVLGLAMSLVAWL-VTTKTEYGVFTVETTGANY
ScDUR3 416 SVGLYYGGISMG-YIYEMMGIIISSAVLPVVLTLCSKDMNLVAAVVSPILGTGLAIMSWL-VCTKSLYKELTVDTTFMDY
AtDUR3 447 AVVLNKAGVSLG-WMYLAMGVLIGSAVIPIAFMLLWSKANAFGAILGATSGCVFGIITWL-TTAKTQYGRVDLDSTGKNG
PiDUR3 426 GVIFFYIGVSMG-WLYTFMGVILGSGVVPIALCITWSKASRAGCVGGAVIGFVVGIAAWLGVTAAYNAGVINVTTSGGNY
CaDur3 415 AIGLYYAKVSMG-YLYEMMGVIIGGAVLSSALTILSKRQNWHAATFTPIISTALAIMAWL-VCTKKKFGSVTYLKTFEDD
OsDur3 452 AVVLNVAGVSLG-WMYLAMGVIVGSAVIPIALLLLWSKANAVGAMGGAVSGCALGVAVWL-TVAKVQYGRVNLDTTGRNA
PutP   390 AANPENRVLGLVSYAWAGFGAAFGPVVL---FSVMWSRMTRNGALAGMIIG-ALTVIVWK------QFGWLGL------Y
hSGLT  442 PIVQSAQSGQLFDYIQSITSYLGPPIAAVFLLAIFWKRVNEPGAFWGLILGLLIGISRMI---TEFAYGTGSCMEPSNCP
NIS    402 AALSSLLGGGVLQGSFTVMGVISGPLLGAFILGMFLPACNTPGVLAGLGAGLALSLWVALGATLYPPSEQTMRVLPSSAA
vSGLT  414 APMLGGIGQAFQ-YIQEYTGLVSPGILAVFLLGLFWKKTTSKGAIIGVVASIPFALFLKF---MPLSMPFMDQMLYTLLF
          .  .  .  .  .  .  .  .  .  .  .
          .  .  .  .  .  .  .  .  .  .  .

UreA   565 ----------------SPSQQQEE---------EEIRKLNKAAFISRCLTVGMVICFLILWPIPMYGSGYVFSKKFFT
ScDUR3 573 DVRVNNEKLIEPNLGVVISNAIFQEDDTQLQNELDEEQRELARGLKIAYFLCVFFALAFLVVWPMPMYGSKYIFSKKFFT
AtDUR3 566 -----------------PAEELREEK--------------LRRAKAWIVKWGLVFTILIVVIWPVLSLPAR-VFSRGYFW
PiDUR3 576 -----------------QDSELDPVG--------------LKKAFKFAAWSSVIMTVIFMILIPLPLFGASTVFSTTGFT
CaDur3 563 ----------------VIASQLVEGEREQYQ----KEKEILNKSFKICCIVCCTLAICLVVLFPMPLYGTKYIFSKKFFT
OsDur3 578 -------------------DELKEER--------------LVHAKRWIVRWGLVFTAVIVVAWPALSLPAR-RYSLGYFT
PutP   459 --------------------------------------------------------------------------------
hSGLT  546 -------------------------------------LLTKPIPDVHLYRLCWSLRNSKEERIDLDAEEENIQEGPKE
NIS    518 ------------------------------------ALADSFYAISYLYYGALGTLTTVLCGALISCLTGPTKRSTLA
vSGLT  503 ----------------------------------------------------------------------INDDDPK
          .  .  .  .  .  .  .  .  .  .  .
          .  .  .  .  .  .  .  .  .  .  .

UreA   619 GWVVVGIIWLFGTAFGVILFPLWEGRSSIKRVGKLMLLD------AMGRQWKSSALVGQGDEESEESGSGAVTPSEKIVA
ScDUR3 653 GWVVVMIIWLFFSAFAVCIYPLWEGRHGIYTTLRGLYWD------LSGQTYKLREWQNSNPQDLHVVTSQISARAHRQSS
AtDUR3 614 FWAIVAIAWGTIGSIVIIGLPLVESWDTIKSVCMGMFTNDRVMKKLDDLNHRLRALTMAVPEAEKIY--LLELEKTKK--
PiDUR3 625 VWVVIGILWTFCSAFAVVLYPLWESREALIMISKGMIQG-YLCQRVSGKFVPADAKEEVPALKGGLMTMIMMVNKLPDHL
CaDur3 623 GWVVVFIIWLFYSAFQVIIFPIWESRTDLYHTFRGIYWD------LTGQTYKLKAWQNEHPEEMHAVRSQLQAQLSRTAV
OsDur3 624 LWAAVAIAWGTVGSVVIILLPVAESWTTITKVCAGMFTNDAVYDRLDDVNLRLRAIMGAMPEAEKRYRQLHETEMHPAGT
PutP   459 ---------FIFGSIGIVVFSLLGKAPSAAM-----------------------------------------------
hSGLT  587 TIEIETQVPEKKKGIFRRAYDLFCGLEQHGAPKMTEEEEKAMKMKMTDTSEKPLWRTVLNVNGIILVTVAVFCHAYFA--
NIS    560 PGLLWWDLARQTASVAPKEEVAILDDNLVKGPEELPTGNKKPPGFLPTNEDRLFFLGQKELEGAGSWTPCVGHDGGRDQQ
vSGLT  510 GISVTSSMFVTDRSFNIAAYGIMIVLAVLYTLFW------------------------------------------
```

Figure S2. Multiple sequence alignment of UreA and homologues of known function *vs.* representative members of the Sodium/solute Symporter Superfamily (SSS). Fully conserved amino acids are shaded in black, structurally conserved amino acids are shaded in grey. Mutated amino acids in UreA by classical or spontaneous mutagenesis are highlighted in blue and red-lined boxes respectively. Dotted vertical lines represent omitted portions of the alignment, which do not bear conserved residues mutated in this work. The proteins included in the alignment are UreA of *Aspergillus nidulans* (GI: 67516273) and its homologues ScDUR3 of *Saccharomyces cerevisiae* (GI: 51013791), AtDUR3 of *Arabidopsis thaliana* (GI: 9758728), PiDUR3 of *Paxillus involutus* (sequence kindly provided by Morel *et a l.*, 2008), CaDUR3 of *Candida albicans* (GI: 68484979), OsDUR3 of *Oryza sativa* (GI: 115483686). SSS members include the sodium/proline symporter, PutP, of *Escherichia coli* (GI: 91210108), the sodium/glucose symporter, hSGLT, of *Homo sapiens* (GI: 4507031), the sodium/iodide symporter, NIS, of *H. sapiens* (GI: 4507035) and the sodium/galactose symporter, vSGLT, of *Vibrio parahaemolyticus* (GI: 8134671).

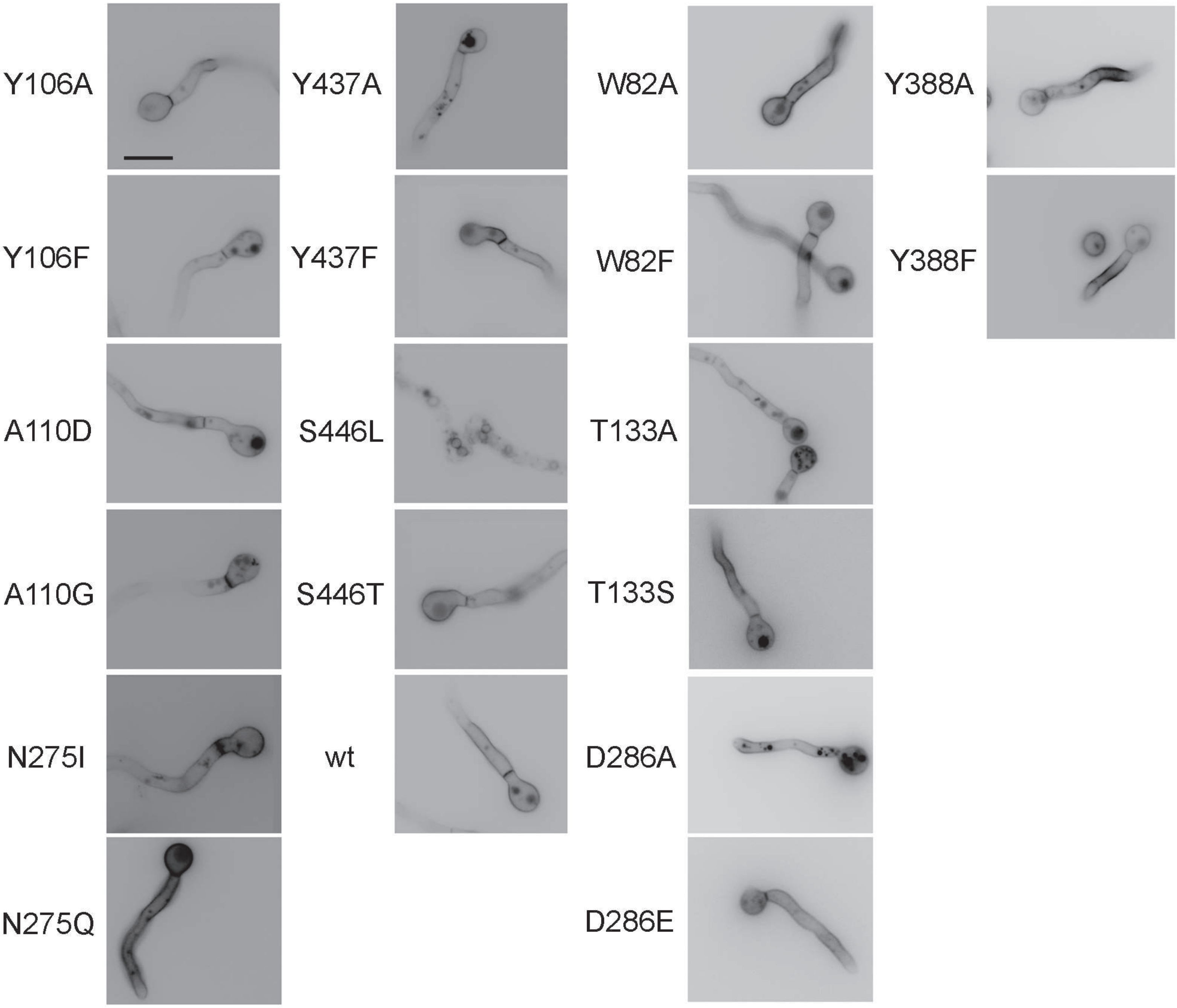

Figure S3. Epifluorescence microscopy of UreA mutants grown in de-repressing conditions. Wild type UreA-GFP localization is shown as control. The bar represents 10 μm.

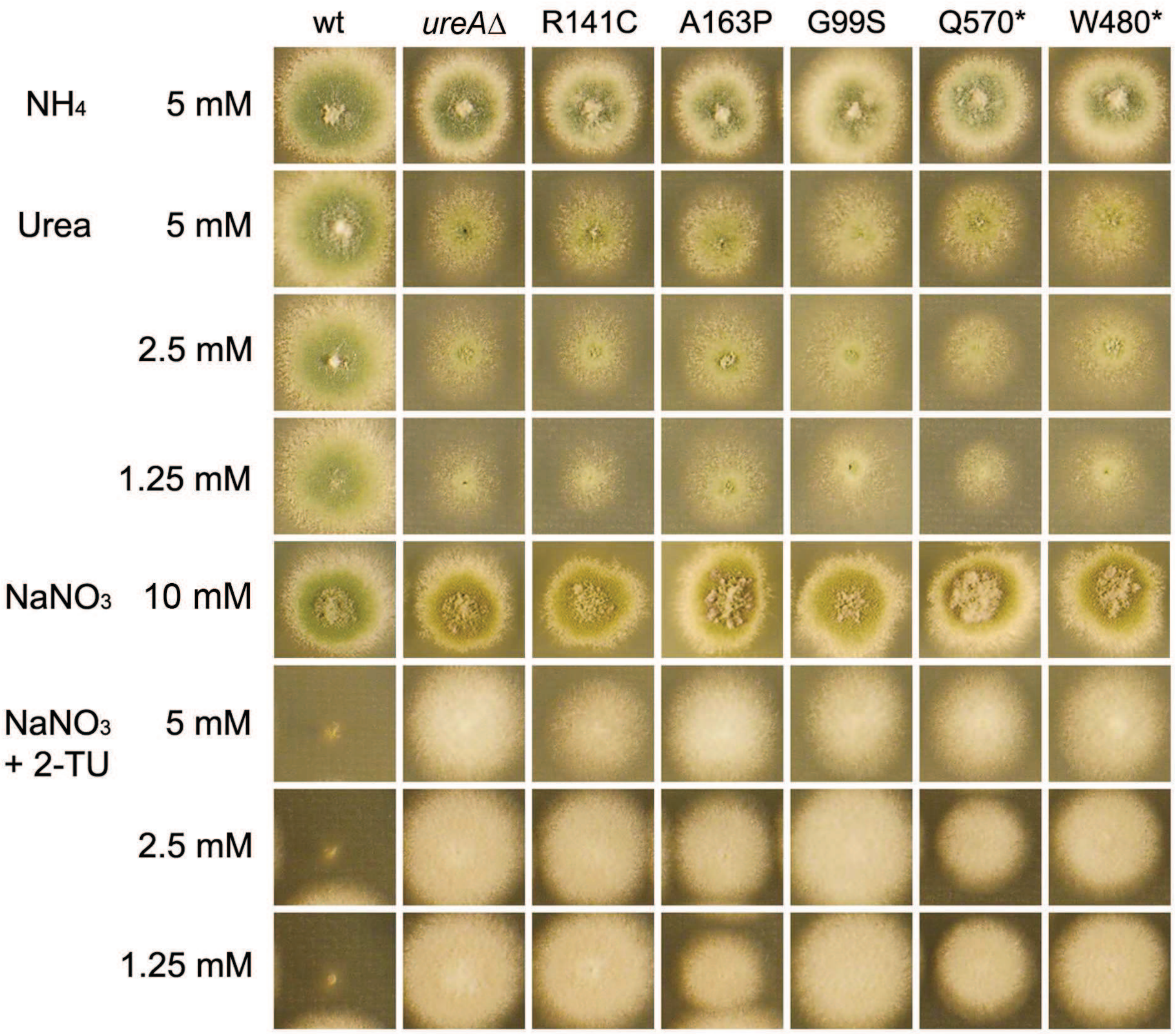

Figure S4. Growth test at 37 ºC of mutant UreA strains isolated by random mutagenesis. Strains were grown on urea as sole nitrogen source or on 2-thiourea to test resistance. In this latter case 10 mM $NaNO_3$ was used as nitrogen source. Growth on ammonium 5 mM or 10 mM $NaNO_3$ was used as a control. A *wt* and a *ureA*Δ strain are shown as positive and negative control, respectively. Similar results were obtained at 25 ºC (data not shown).

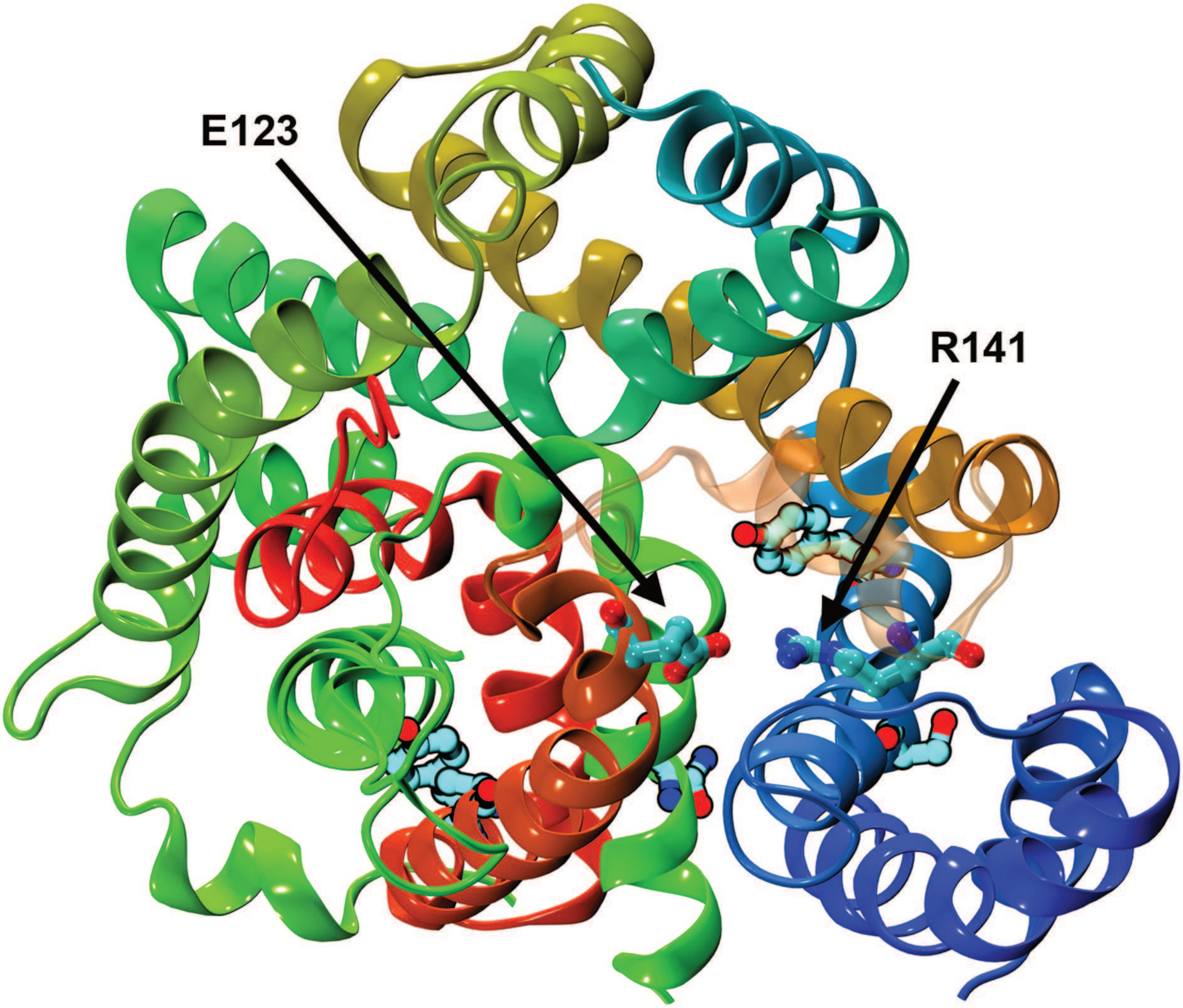

Figure S5. Molecular representation of the UreA model in the conformation presenting an outward facing cavity seen from the extracellular side. R141 and E123, which can form a salt bridge are indicated and shown in glossy color. ICH3/4 is shown as semitransparent in orange.

Table S1. *Aspergillus nidulans* strains used and constructed during this study.

Strain	***Genotype***
MVD 001	*pabaA1 veA1*
P002	*yA2 pabaA1*
MVD 10A	*ureA::gfp::AFpyrG riboB2 pyrG89 pyroA4 ∆nkuA::argB veA1*
MVD 13A	*ureA∆::riboB riboB2 pyrG89 pyroA4 ∆nkuA::argB veA1*
MVD 14A	*yA2 ureA∆::riboB riboB2 pyrG89 pyroA4 ∆nkuA::argB veA1*
MVD 100	*ureA1 biA1 veA1*
MVD 103	*ureA905 pabaA1 veA1*
MVD 205	*wA3 yA2 pabaA1 hhoA::mrfp::pyrGAf*
MVD 105*	*yA2 ureA1::gfp::AFpyrG pyrG89 pyroA4 riboB2 nkuA::argB veA1*
MVD 106*	*yA2 ureA905::gfp::AFpyrG pyrG89 pyroA4 riboB2 nkuA::argB veA1*
W82A*	*yA2 ureAW82A::gfp::AFpyrG pyrG89 pyroA4 riboB2 nkuA::argB veA1*
W82F*	*yA2 ureAW82F::gfp::AFpyrG pyrG89 pyroA4 riboB2 nkuA::argB veA1*
Y106A*	*ureAY106A::gfp::AFpyrG pyrG89 pyroA4 riboB2 nkuA::argB veA1*
Y106F*	*ureAY106F::gfp::AFpyrG pyrG89 pyroA4 riboB2 nkuA::argB veA1*
A110D*	*ureAA110D::gfp::AFpyrG pyrG89 pyroA4 riboB2 nkuA::argB veA1*
A110G*	*ureAA110G::gfp::AFpyrG pyrG89 pyroA4 riboB2 nkuA::argB veA1*
T133A*	*yA2 ureAT133A::gfp::AFpyrG pyrG89 pyroA4 riboB2 nkuA::argB veA1*
T133S*	*ureAT133S::gfp::AFpyrG pyrG89 pyroA4 riboB2 nkuA::argB veA1*
N275I*	*ureAN275I::gfp::AFpyrG pyrG89 pyroA4 riboB2 nkuA::argB veA1*
N275Q*	*ureAN275I::gfp::AFpyrG pyrG89 pyroA4 riboB2 nkuA::argB veA1*
D286A*	*yA2 ureAD286A::gfp::AFpyrG pyrG89 pyroA4 riboB2 nkuA::argB veA1*
D286E*	*yA2 ureAD286E::gfp::AFpyrG pyrG89 pyroA4 riboB2 nkuA::argB veA1*
Y388A*	*ureAY388A::gfp::AFpyrG pyrG89 pyroA4 riboB2 nkuA::argB veA1*
Y388F*	*ureAY388F::gfp::AFpyrG pyrG89 pyroA4 riboB2 nkuA::argB veA1*
Y437A*	*ureAY437A::gfp::AFpyrG pyrG89 pyroA4 riboB2 nkuA::argB veA1*
Y437F*	*ureAY437F::gfp::AFpyrG pyrG89 pyroA4 riboB2 nkuA::argB veA1*
S446L*	*ureAS446L::gfp::AFpyrG pyrG89 pyroA4 riboB2 nkuA::argB veA1*
S446T*	*ureAS446T::gfp::AFpyrG pyrG89 pyroA4 riboB2 nkuA::argB veA1*
MVD 313*	*ureAS446L::gfp::AFpyrG hhoA::mrfp::AFpyrG pabaA1 (pyrG89) veA1*

* Obtained in this study.

Table S2. Oligonucleotides used in this study

Primer name	*5´to 3´Sequence*
Ure5-F	GAAACCTGGAGCAGTCGAAG
Ure3-R	CCCGATTTCTGAGACAAGGA
Ure5-N	GCACCGATGACAAGGGAGAT
Ure3-N	ACCAATGGATCTGGCACTAAAC
W82A-F	GCTGTCGTGAGCAGTGCTACCTGGGCAGCTACTCTGCTG
W82A-R	GAGTAGCTGCCCAGGTAGCACTGCTCACGACAGCAGAGG
W82F-F	GCTGTCGTGAGCAGTTTCACCTGGGCAGCTACTCTGCTG
W82F-R	AGAGTAGCTGCCCAGGTGAAACTGCTCACGACAGCAGAG
Y106A-F	CCTCGGGGCCGTTCTTCGCAGCATCGGGTTGGTCTTC
Y106A-R	GAAGACCAACCCGATGCTGCGAAGAACGGCCCCGAGG
Y106F-F	CCTCGGGGCCGTTCTTCTTTGCATCGGGTTGGTCTTC
Y106F-R	GAAGACCAACCCGATGCAAAGAAGAACGGCCCCGAGG
A110D-F	GACAGTTTGCAGGCGACACCGTTCAGATCATC
A110D-R	GATGATCTGAACGGTGTCGCCTGCAAACTGTC
A110G-F	ACTGACAGTTTGCAGGCGGCACCGTTCAGATCATCTTG
A110G-R	CAAGATGATCTGAACGGTGCCGCCTGCAAACTGTCAGT
T133A-F	CGCCTAACGCACACGCATTCCTGGAAGCCATCCGTG
T133A-R	TGGCTTCCAGGAATGCGTGTGCGTTAGGCGCGCGTC
T133S-F	CGCCTAACGCACACTCATTCCTGGAAGCCATCCGTG
T133S-R	TGGCTTCCAGGAATGAGTGTGCGTTAGGCGCGCGTC
N275I-F	ATCTTCTGGGTCATCATCCTCGTCGGTAACTTC
N275I-R	GAAGTTACCGACGAGGATGATGACCCAGAAGAT
N275Q-F	GTATCTTCTGGGTCATCCAGCTCGTCGGTAACTTCG
N275Q-R	CGAAGTTACCGACGAGCTGGATGACCCAGAAGATAC
D286A-F	GCACTGTCTTCCTGGCCAACGGCTACTACAACAAGG
D286A-R	TTGTTGTAGTAGCCGTTGGCCAGGAAGACAGTGCCGAAG
D286E-F	GCACTGTCTTCCTGGAGAACGGCTACTACAACAAGG
D286E-R	TTGTTGTAGTAGCCGTTCTCCAGGAAGACAGTGCCGAAG
Y388A-F	GTGTCCTCGATCCTCACCGCCGATATCTACCAAGCCTAC
Y388A-R	TAGGCTTGGTAGATATCGGCGGTGAGGATCGAGGACAC
Y388F-F	TGTCCTCGATCCTCACCTTCGATATCTACCAAGCCTA
Y388F-R	TAGGCTTGGTAGATATCGAAGGTGAGGATCGAGGACA
Y437A-F	CGGTATGGGCTATCTCGCTCTTCTCATGGGCGTC
Y437A-R	GACGCCCATGAGAAGAGCGAGATAGCCCATACCG
Y437F-F	GTATGGGCTATCTCTTCCTTCTCATGGGCG
Y437F-R	CGCCCATGAGAAGGAAGAGATAGCCCATAC
S446L-F	GGCGTCATCATCTCCTTAGCCGTGTTCCCGGGC
S446L-R	GCCCGGGAACACGGCTAAGGAGATGATGACGCC
S446T-F	GGCGTCATCATCTCCACAGCCGTGTTCCCGG
S446T-R	CCGGGAACACGGCTGTGGAGATGATGACGCC

Mutated codons are underlined. Modified bases are denoted in black.

Table S3. Summary of modelling data

	Template (PDB id)	*Identity, E and p values*	*Ramachandran plot statistics*	*Averaged G-Factors**
Inward Facing	3DH4	18 %<br>3,77E^{-65}<br>1.2E^{-69}	a.a. most favored regions: 91.7 %<br>a.a. in allowed regions:7.8 %<br>a.a. in disallowed regions:0.5 %	Psi-psi: 0.28<br>Chi1-Chi2: 0.09<br>Chi1 only: 0.12<br>Omega: -0.38
Outward Facing	2JLN	14%<br>5.6 E^{-09}<br>1.8E^{-13}	a.a. most favored regions: 92.1 %<br>a.a. in allowed regions:7.7 %<br>a.a. in disallowed regions:0.2 %	Psi-psi: 0.26<br>Chi1-Chi2: -0.6<br>Chi1 only: 0.25<br>Omega: -0.34

* G-factors are a measure of unusual values for stereo chemical properties. Very negative values are indicative of significant deviations from standard conformations.
